# Treatment of Silylene–Phosphinidene with Chalcogens Resulted Exclusively in the Formation of Silicon‐Bonded Chalcogens

**DOI:** 10.1002/chem.201902661

**Published:** 2019-08-13

**Authors:** Soumen Sinhababu, Mujahuddin M. Siddiqui, Samir Kumar Sarkar, Annika Münch, Regine Herbst‐Irmer, Anjana George, Pattiyil Parameswaran, Dietmar Stalke, Herbert W. Roesky

**Affiliations:** ^1^ Institut für Anorganische Chemie Universität Göttingen Tammannstrasse 4 37077 Göttingen Germany; ^2^ Department of Chemistry National Institute of Technology Calicut NIT Campus P.O., Kozhikode 673601 Kerala India

**Keywords:** chalcogens, phosphinidenes, phosphorus, silicon, silylene–phosphinidene

## Abstract

Chalcogen‐bonded silicon phosphinidenes LSi(E)−P−^*Me*^cAAC (E=S (**1**); Se (**2**); Te (**3**); L=PhC(N*t*Bu)_2_; ^*Me*^cAAC=C(CH_2_)(CMe_2_)_2_N‐2,6‐*i*Pr_2_C_6_H_3_)) were synthesized from the reactions of silylene–phosphinidene LSi−P−^*Me*^cAAC (**A**) with elemental chalcogens. All the compounds reported herein have been characterized by multinuclear NMR, elemental analyses, LIFDI‐MS, and single‐crystal X‐ray diffraction techniques. Furthermore, the regeneration of silylene–phosphinidene (**A**) was achieved from the reactions of **2**–**3** with L′Al (L′=HC{(CMe)(2,6‐*i*Pr_2_C_6_H_3_N)}_2_). Theoretical studies on chalcogen‐bonded silicon phosphinidenes indicate that the Si−E (E=S, Se, Te) bond can be best represented as charge‐separated electron‐sharing σ‐bonding interaction between [LSi−P−^*Me*^cAAC]^+^ and E^−^. The partial double‐bond character of Si−E is attributed to significant hyperconjugative donation from the lone pair on E^−^ to the Si−N and Si−P σ*‐molecular orbitals.

Phosphinidenes (R−P) are phosphorus analogues of carbenes and nitrenes.^1^ Previously, phosphinidenes were assumed as short‐lived intermediates, which were observed spectroscopically in the gas phase and in matrices.^2^ In 1975, Lorenz and co‐workers isolated the first structurally characterized stable phosphinidene complex stabilized in the coordination sphere of a transition metal.^3^ After that, several other groups reported transition‐metal–phosphinidene complexes, in which the phosphinidene acts as ligand.^4^ In comparison with the transition‐metal complexes of phosphinidenes, the main‐group‐element phosphinidene complexes are limited. Our group has synthesized a cyclic (alkyl)(amino)carbene (cAAC)‐anchored silylene–phosphinidene (**A**, Scheme 1) through a two‐step synthetic route starting from a heteroleptic silylene monochloride and a chlorophosphinidene.^5^ Recently we isolated a silylene, stabilized through two terminal phosphinidene ligands (**B**, Scheme 2).^6^ Another interesting example of a silylene with a low‐coordinate phosphorus atom is the NHC‐stabilized phosphasilenylidene (**C**, Scheme 1) that was synthesized by Filippou and co‐workers.^7^ Furthermore, we reported cAAC–dichlorosilylene‐stabilized phosphinidene (^*Cy*^cAAC)SiCl_2_→P−Tip (**D**, Scheme 1) starting from ^*Cy*^cAAC, HSiCl_3_, and TipPCl_2_ (Tip=2,4,6‐*i*Pr_2_C_6_H_2_).^8^ Further reduction of (^*Cy*^cAAC)SiCl_2_→P−Tip (**D**, Scheme 1) with sodium napthalenide resulted in the dimeric heavier analogue of ketenimine‐containing phosphorus and silicon atoms [(^*Cy*^cAAC)Si(P−Tip)]_2_ (**E**, Scheme 1).^9^ From the aforementioned discussion it is clear that few silylene–phosphinidene complexes are known, although reactivity studies on those complexes are yet to be explored. Herein we report that the silylene–phosphinidene complex (**A**) reacts smoothly with elemental chalcogens to form the chalcogen‐bonded silicon phosphinidenes LSi(E)−P−^*Me*^cAAC (E=S (**1**); Se (**2**); Te (**3**)).

**Scheme 1:**
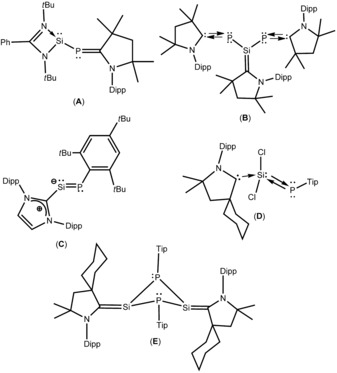
Examples of structurally characterized silylene–phosphinidene (**A**–**E**).

**Scheme 2:**
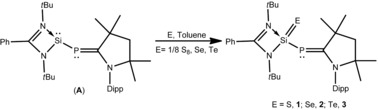
Synthesis of compounds **1**–**3**.

The chemistry of compounds with double bonds between silicon and chalcogens are of great interest because they are heavier congeners of ubiquitous ketones.^10^ Several stable silicon–chalcogen species containing double bonds have been developed using kinetic protection from the bulky ligand and/or thermodynamic stabilization from the Lewis donor as well as from the acceptor. Utilizing kinetic and thermodynamic protection, several examples of compounds containing a silicon–chalcogen double bond have been reported, by the groups of West, Kira, Driess, Filippou and others.^11^ Driess and co‐workers described the donor‐stabilized thiosilanoic phosphane L′Si(S)PH_2_, which is the only example of a compound with a silicon–chalcogen double bond and a phosphine functionality.^12^ In this manuscript, we report for the first time the successful synthesis of LSi(E)−P−^*Me*^cAAC (E=S (**1**); Se (**2**); Te (**3**)) with phosphinidene functionality. Compounds **1**–**3** were characterized by single‐crystal X‐ray structural investigation and multinuclear NMR spectroscopy. An equimolar reaction of compound **A** with elemental sulfur and selenium at room temperature in toluene afforded compounds **1** and **2** in 68 % and 75 % yield, respectively (Scheme 2). A 1:1:1 reaction of **A** with elemental tellurium in toluene at 60 °C for 12 h yielded LSi(Te)−P−^*Me*^cAAC (**3**) in 79 % yield (Scheme 2, for details see the Supporting Information). In catalytic processes, the regeneration of parent molecules are essential steps through reductive elimination. In this issue, recovering a Si^II^ compound from its comparatively stable Si^IV^ compound under mild condition is considerably challenging. With this in mind, we reacted **1** with monomeric Al^I^ (L′Al, L′=HC{(CMe)(2,6‐*i*Pr_2_C_6_H_3_N)}_2_) at room temperature as well as at elevated temperature, but no chalcogen transfer occurred. Nonetheless, **2** and **3** react with L′Al at 60 °C, resulting in the formation of parent silylene–phosphinidene (**A**, Scheme 3, for details see the Supporting Information).

**Scheme 3:**
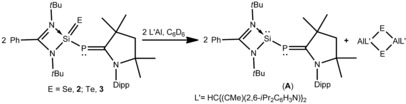
Regeneration of **A** from **2**–**3**.

Compounds **1**–**3** are thermally stable with melting points over 200 °C, but they are sensitive towards moisture. They are fully characterized by NMR spectroscopy, elemental analyses, LIFDI‐MS, and X‐ray single‐crystal structure analysis. The ^31^P{^1^H} NMR spectra of **1**–**3** exhibit a singlet at *δ*=+2.33, +9.37, and +21.67 ppm, respectively, which are highfield shifted compared with the starting material, silylene–phosphinidene (**A**). In the ^29^Si{^1^H} NMR spectra, **1**–**3** display doublets at *δ*=+20.48 (^1^ *J*_Si−P_=107 Hz), +16.96 (^1^ *J*_Si−P_=117 Hz), and −10.94 ppm (^1^ *J*_Si−P_=127 Hz), respectively, due to the coupling with the phosphorus atom. The difference of the chemical shift between **1**–**3** and **A** in the ^29^Si{^1^H} NMR spectra is due to the different silicon oxidation states (+4 and +2). The ^77^Se{^1^H} NMR spectrum of **2** shows a doublet at *δ*=−286.13 ppm (^2^ *J*_Se−P_=18 Hz) due to the coupling with the phosphorus atom. For the same reason the ^125^Te{^1^H} NMR spectrum of **3** exhibits a doublet at *δ*=−835.45 ppm (^2^ *J*_Te−P_=38 Hz). The LIFDI mass spectra of **1**–**3** in toluene exhibit molecular‐ion peaks at 607.4, 655.3, and 705.4, respectively.

Single crystals of **1**–**3** suitable for X‐ray structural analysis were obtained from toluene solution either at 0 °C or at room temperature (for details see the Supporting Information). Compounds **1**–**3** crystallize in the monoclinic space group *P*2_**1**_/*c*. All three structures are isostructural, whereas **1** and **2** are even isomorphous. Compound **3** crystallizes as a pseudo‐merohedral twin with two molecules in the asymmetric unit. As a representative for all the molecular structure of **1** is depicted in Figure 1. It reveals the Si atom to be fourfold coordinated, adopting a distorted tetrahedral geometry. The amidinate ligand is bound in a N,N′ chelating fashion with two rather different Si−N bond lengths. Formation of the Si−E bonds is accompanied by a decrease in the Si−N as well as Si−P bond length. The Si−N bond lengths of **1**, **2**, and **3** are about 3 pm shorter than in **A** (Table 1). Similarly, the Si−P bond length in **1**, **2**, and **3** is shortened by 4 pm compared with **A**. The Si−S bond length in **1** and Si−Se bond length in **2** are slightly longer than those in [{PhC(N*t*Bu)_2_}Si(S)N(SiMe_3_)_2_] and [{PhC(N*t*Bu)_2_}Si(Se)N(SiMe_3_)_2_] (1.987(8) and 2.136(9) Å, respectively).11h The Si−Te bond length in **3** is similar to the one observed in [{PhC(N*t*Bu)_2_}Si(Te)N(SiMe_3_)_2_], (2.3720(15) Å).11i The Si−E bond lengths in **1**–**3** are well within the range of previous reported Si−E double bond lengths.

**Figure 1:**
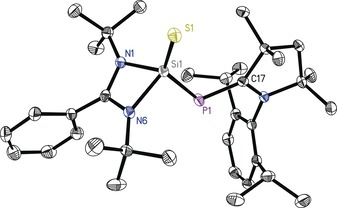
Molecular structure of LSi(S)−P−^*Me*^cAAC (**1**). The anisotropic displacement parameters are depicted at the 50 % probability level. Hydrogen atoms are omitted for clarity.

**Table 1:** Selected bond lengths [Å] and bond angles [°] of **1**–**3** and **A**.

	**1**	**2**	**3**	**A** 5
Si−N	1.8480(14)	1.8415(12)	1.845(2)	1.8864(15)
	1.8366(14)	1.8346(11)	1.829(2)	1.8751(15)
			1.850(2)	
			1.837(2)	
Si−P	2.2433(7)	2.2384(7)	2.2395(11)	2.2970(7)
			2.2515(12)	
Si−E	2.0018(7)	2.1404(5)	2.3766(8)	
			2.3849(8)	
				
E‐Si‐N	119.30(5)	118.40(4)	114.35(8)	
	117.10(5)	116.49(4)	112.54(8)	
E‐Si‐P	135.11(3)	136.00(2)	135.97(4)	
			136.56(4)

Quantum‐mechanical calculations were performed at the M06/def2‐TZVPP//BP86/def2‐SVP level of theory to understand the electronic structure and reactivity of silylene–phosphinidene **A** as well as chalcogen‐bonded silicon phosphinidenes **1**, **2**, and **3**.^13^ The molecular electrostatic potential (MESP) map of silylene–phosphinidene **A** is given in Figure S2.3a (Supporting Information). The ESP value in the direction of the lone pair at P (−35.5 kcal mol^−1^) is slightly higher than that in the direction of the lone pair at Si (−30.8 kcal mol^−1^). This indicates a slightly higher nucleophilicity of the first than the second (Table S2.1). The occupancy of the P (1.943 e) and Si (1.925 e) lone pair in **A** are well corroborated with ESP values (Table S2.2). This is in accordance with our previous theoretical study on silylene–phosphinidenes.^5^

Although the nucleophilicity of the phosphorus atom is slightly higher than that of the silicon atom in **A**, the reactions of silylene–phosphinidene **A** with chalcogens result in silicon bonded chalcogens in the phosphinidenes **1**, **2**, and **3**. To account for this observation, we have calculated the reaction energies for the formation of chalcogen‐bonded silicon phosphinidenes **1**, **2**, and **3** as well as hypothetical chalcogen‐bonded phosphorus phosphinidenes **1′**, **2′**, and **3′** (Figure S2.2, Table S2.4, Supporting Information). All the reaction energies are exothermic, and the energies become more positive with the descent from sulfur to tellurium. The reaction energies for the formation of silicon‐bonded chalcogen phosphinidenes **1** (−107.3), **2** (−88.2), and **3** (−71.2 kcal mol^−1^) are much higher than chalcogen‐bonded phosphorus phosphinidenes **1′** (−77.0), **2′** (−59.4), and **3′** (−46.8 kcal mol^−1^; Table S2.4). Hence the formation of the P−E bond is less favorable than the formation of a Si−E bond. Note that our calculated reaction energies are comparable to the previously reported bond‐dissociation energies for Si=S (112.8), Si=Se (95.5), and Si=Te (77.2 kcal mol^−1^) bonds in CH_3_Si(=E)OH compounds (E=S, Se, Te) at the MP2/6–311++G(2df, 2pd)//MP2/6–311+G(d, p) level of theory.^14^ The Si−S bond is the strongest among Si−E bonds in **1**–**3**, which is in accordance with the experimental observation that the regeneration of parent molecule **A** could not be achieved when **1** is treated with the L′Al (L′=HC{(CMe)(2,6‐*i*Pr_2_C_6_H_3_N)}_2_) (Scheme 3). In contrast, this is feasible when **2** and **3** are reacted with L′Al at 60 °C.

The calculated geometrical parameters of **1**–**3** at the BP86/def2‐SVP level of theory are close to those from the experimental geometries (Figure S2.1, Supporting Information). The Si−E bond lengths in **1** (2.019), **2** (2.160), and **3** (2.398 Å) in turn are close to those of previously reported Si=E double bond lengths.^11^ Note that the Si−S bond length in **1** is comparable to those in the amidinate stabilized siladithiocarboxylate (2.030 Å),^15^ as well as to silanethione (2.013 Å).^16^ The partial double‐bond character in the Si−E bonds in **1**–**3** is in agreement with the Wiberg bond index of Si−E bond (1.48, 1.53, and 1.54, Table 2). The natural‐charge analysis indicates that the polarity of the Si−E bond decreases when E changes from S to Te. This is in corroboration with MESP data in which the global minimum of the ESP is observed near to the chalcogen (Figure S2.3) and the corresponding ESP value decreases when E changes from S to Te. The ESP values near to chalcogen are −38.9 (**1**), −36.1(**2**), and −32.0 kcal mol^−1^ (**3**).

**Table 2:** The natural charge (*q*) and Wiberg bond orders (*p*) at the M06/def2‐TZVPP//BP86/def2‐SVP level of theory.

	**A**	**1**	**2**	**3**
*q*(E)	–	−0.82	−0.70	−0.53
*q*(Si)	0.76	1.37	1.26	1.09
*p*(Si−E)	–	1.48	1.53	1.54

The EDA‐NOCV analysis was carried out to further shed light on the nature of the Si−E bond. Two different bonding situations were analyzed, and the results are given in Table S2.5 (Supporting Information). The first bonding interaction represents charge separated electron‐sharing interaction between [LSi−P−^*Me*^cAAC]^+^ and E^−^. The second bonding interaction represents donor–acceptor interaction between [LSi−P−^*Me*^cAAC] and E. The best bonding representation is the one having the least value for orbital stabilization energy Δ*E*_orb_.^13^ Δ*E*_orb_ for the charge separated electron sharing interaction in **1**, **2**, and **3** are −215.8, −189.1, and −159.6 kcal mol^−1^, respectively. The Δ*E*_orb_ for the donor–acceptor interaction in **1**, **2**, and **3** are −265.2, −214.7, and −164.9 kcal mol^−1^, respectively. Hence the best bonding description for the Si−E bond in **1**–**3** is the charge‐separated electron‐sharing interaction between the fragments. However, the difference in the Δ*E*_orb_ between the two bonding models in **3** is only 5.3 kcal mol^−1^. Hence, the donor–acceptor interaction also contributes towards the ground electronic structure of Si−Te bond in **3**.

The complete EDA‐NOCV results for the best possible bonding interaction are given in Table S2.6 in the Supporting Information. The electrostatic interaction has a greater contribution (53.0 % in **1**, 55.3 % in **2**, 56.5 % in **3**) in stabilizing the Si−E bond than the orbital interaction. The magnitude of electrostatic interaction decreases from **1** (−243.6) to **2** (−233.6) to **3** (−207.0 kcal mol^−1^). This is in agreement with the natural charges (Table 2) on Si and E in **1**–**3**. The analysis of components of the interaction energy indicates that the electron sharing Si^+^−E^−^ (Δ*E*_1_, Table S2.6) contributes 64.7–66.4 % of the total orbital‐interaction energy. The hyperconjugative donation from the lone pairs at E^−^ to the Si−N as well as Si−P σ*‐MOs also contributes significantly to the orbital‐interaction energy. The deformation density plots corresponding to these hyperconjugative interactions in **1** are depicted in Figure 2. Similar deformation‐density plots are observed for compounds **2** and **3** as well (Figure S2.4). The strength of these stabilizing interactions decreases when the element E changes from S to Te (Δ*E*_2_+Δ*E*_3_; −54.0 in **1**, −46.9 in **2**, and −38.4 kcal mol^−1^ in **3**; Table S2.6, Figure S2.4). Hence, the partial multiple‐bond nature of Si−E bond is attributed to the hyperconjugative donation of the lone pairs at E^−^ to the Si−N and Si−P σ*‐MOs.

**Figure 2:**
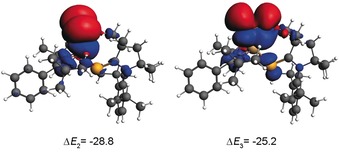
Plots of deformation densities (BP86/TZ2P) corresponding to the hyperconjugative donation from the lone pairs at E^−^ to the Si−N and Si−P σ*‐MOs in **1**. The direction of charge flow is from red to blue. The isosurface value for the plot is 0.0003 electrons per cubic bohr.

In summary, the reaction of silylene–phosphinidene with the heavier chalcogens S, Se, and Te resulted in the selective formation of the first silicon‐bonded chalcogen phosphinidenes (**1**–**3**). All the compounds were fully characterized using multinuclear NMR, LIFDI‐MS, X‐ray crystallography, and theoretical calculations. The theoretical calculations confirmed the oxidation of silylene to be more favorable than that of the phosphinidene. The parent silylene–phosphinidene **A** was regenerated by the reaction of **2** and **3** with L′Al (L′=HC{(CMe)(2,6‐*i*Pr_2_C_6_H_3_N)}_2_). Also, the dichotomy of regeneration of **A** by **2** and **3** only was rationalized by theoretical calculations which suggested that the Si−S bond is the strongest among the Si−E bonds in **1**–**3**. The hyperconjugative donation from the lone pair on E^−^ to the Si−N and Si−P σ*‐molecular orbitals induces a partial double‐bond character to the Si−E bond.

## Experimental Section

The datasets were collected on an Incoatec Mo Microsource^17^ (**3**) and on a Bruker TXS‐Mo rotating anode (**1**, **2**) with mirror optics and an APEX II detector with a D8 goniometer. The data were integrated with SAINT.^18^ A multi‐scan absorption correction and a 3 λ correction^19^ was applied using SADABS.^20^ The structures were solved by SHELXT^21^ and refined on F^2^ using SHELXL^22^ in the graphical user interface ShelXle.^23^ Crystal data at 100(2) K for **1**: C_35_H_54_N_3_PSSi, *M_r_*=607.93 g mol^−1^, 0.111×0.245×0.271 mm, monoclinic, *P*2_1_/*c*, *a=*13.870(3), *b=*10.073(2), *c=*26.585(3) Å, *β*=101.65(2)*°, V=*3637.7(12) Å^3^, *Z=*4, *μ* (Mo K_α_)=0.192 mm^−1^, *θ*_max_=27.50°, 54 419 reflections measured, 8340 independent (*R_int_=*0.0627), *R*_1_ *=*0.0387 [*I*>2σ(*I*)], *wR*_2_ *=*0.0973 (all data), res. density peaks: 0.324 to −0.251 e Å^−3^; Crystal data for **2**: C_35_H_54_N_3_PSeSi, *M_r_*=654.83 g mol^−1^, 0.351×0.257×0.145 mm, monoclinic, *P*2_1_/*c*, *a=*13.903(2), *b=*10.077(2), *c=*26.651(3) Å, *β*=102.18(3)*°, V=*3649.8(11) Å^3^, *Z=*4, *μ* (Mo K_α_)=1.131 mm^−1^, *θ*_max_=28.34°, 125 128 reflections measured, 9105 independent (*R_int_=*0.0424), *R*_1_ *=*0.0252 [*I*>2σ(*I*)], *wR*_2_ *=*0.0640 (all data), res. density peaks: 0.406 to −0.182 e Å^−3^; Crystal data for **3**: C_35_H_54_N_3_PTeSi, *M_r_*=703.47 g mol^−1^, 0.254×0.119×0.082 mm, monoclinic, *P*2_1_/*c*, *a=*35.937(3), *b=*9.237(2), *c=*23.498(3) Å, *β*=109.09(2)*°*, *V=*7371(2) Å^3^, *Z=*8, *μ* (Mo K_α_)=0.909 mm^−1^, *θ*_max_=25.32°, 120 742 reflections measured, 13 421 independent (*R_int_=*0.0522), R_1_ *=*0.0259 [*I*>2σ(*I*)], *wR*_2_ *=*0.0500 (all data), res. density peaks: 0.570 to −0.559 e Å^−3^.

CCDC 1891853 (**1**), 1891854 (**2**), and 1891855 (**3**) contain the supplementary crystallographic data for this paper. These data are provided free of charge by The Cambridge Crystallographic Data Centre.

## Conflict of interest

The authors declare no conflict of interest.

## Supporting information

As a service to our authors and readers, this journal provides supporting information supplied by the authors. Such materials are peer reviewed and may be re‐organized for online delivery, but are not copy‐edited or typeset. Technical support issues arising from supporting information (other than missing files) should be addressed to the authors.

Supplementary
