# Supplementary material for: Treatment of Silylene–Phosphinidene with Chalcogens Resulted Exclusively in the Formation of Silicon‐Bonded Chalcogens
Source: Chemistry. 2019 Aug 13;25(49):11422–6. doi: 10.1002/chem.201902661 (PMC6771779; doi:10.1002/chem.201902661)
Supplement: Supplementary file 1 — Supplementary [file CHEM-25-11422-s001.pdf]

CHEMISTRY
A European Journal

Supporting Information

# Treatment of Silylene–Phosphinidene with Chalcogens Resulted Exclusively in the Formation of Silicon-Bonded Chalcogens

Soumen Sinhababu,[a] Mujahuddin M. Siddiqui,[a] Samir Kumar Sarkar,[a] Annika Münch,[a] Regine Herbst-Irmer,[a] Anjana George,[b] Pattiyil Parameswaran,*[b] Dietmar Stalke,*[a] and Herbert W. Roesky*[a]

chem_201902661_sm_miscellaneous_information.pdf

# Treatment of Silylene-Phosphinidene with Chalcogens Resulted Exclusively in the Formation of Silicon Bonded Chalcogens

Soumen Sinhababu,[a] Mujahuddin M. Siddiqui,[a] Samir Kumar Sarkar,[a] Annika Münch,[a] Regine Herbst-Irmer,[a] Anjana George, [b] Pattiyil Parameswaran,*[b] Dietmar Stalke,*[a] and Herbert W. Roesky*[a]

# Supporting Information

**Content:**

**S1**. Experimental Section (2-15)

**S2**. Theoretical analysis (16-33)

**S3**. X-Ray Crystallographic Analysis (33-44)

**S4**. References (45-47)

## S1. Experimental Section

All manipulations were performed under a dry argon or nitrogen atmosphere using either Schlenk line or glovebox techniques. Solvents were dried by refluxing with sodium/potassium under dry $N_2$ prior to use. $C_6D_6$ was dried by stirring for 2 days over Na/K alloy followed by distillation and degassed. NMR ($^{1}H$, $^{13}C$, $^{29}Si$, $^{31}P$, $^{77}Se$ and $^{125}Te$) spectra were recorded on a Bruker Avance II 500 MHz spectrometer. Melting points of the new compounds were recorded in a sealed glass capillary using the Büchi-540 instrument. Elemental analyses were performed by the Analytisches Labor des Instituts für Anorganische Chemie der Universität Göttingen. LIFDI spectrometries were carried out on a Joel AccuTOF spectrometer under inert atmosphere. LSi-P-$^{Me}$cAAC (**A**) was prepared according to the literature procedure.[1]

**LSi(S)-P-$^{Me}$cAAC (1):** Compound **A** (400 mg, 0.69 mmol) and elemental sulfur (22 mg, 0.69 mmol) were placed in a Schlenk flask and toluene (20 ml) was added at room temperature. Then the reaction mixture was stirred for 12 h at room temperature. During that time the color of the

reaction mixture changes from orange red to yellow. After filtration, the filtrate was reduced to 3 mL and stored at 0 °C. After 1 day, compound **1** was obtained as light yellow crystals (287 mg, 68%). Mp: 245 °C. $^{1}H$ NMR (500 MHz, $C_6D_6$, 298 K, ppm): $\delta$ = 1.02 (s, 6 H, C$H_3$), 1.24 (d, 6 H, CH$Me_2$), 1.28 (s, 18 H, t-Bu), 1.65 (d, 6 H, CH$Me_2$), 1.84 (s, 2 H, C$H_2$), 2.40 (s, 6 H, C$H_3$), 3.04 (sept, 2 H, C$H$Me$_2$), 6.58−7.11 (m, 8 H, $C_6H_3$ and $C_6H_5$). $^{13}C\{^{1}H\}$ NMR (125.8 MHz, $C_6D_6$, 298 K, ppm): $\delta$ = 24.27, 27.71, 27.78, 29.19, 29.46, 31.46, 32.96, 51.97, 52.06, 54.91, 56.34, 70.12, 125.21,127.71, 127.79, 127.97, 128.16, 128.34, 129.18, 129.30, 129.82, 131.54, 147.89, 172.15, 221.42 (d, $^{1}J_{CP}$ = 74 Hz). $^{31}P\{^{1}H\}$ NMR (202 MHz, 298 K, $C_6D_6$) $\delta$ +2.33; $^{29}Si\{^{1}H\}$ NMR (99 MHz, 298 K, $C_6D_6$, ppm) δ= +20.48 ppm ($^{1}J_{SiP}$ = 107 Hz). Anal. Calcd (%) for $C_{35}H_{54}N_3PSSi$ (Mw = 607.96): C, 69.15; H, 8.95; N, 6.91. Found: C, 68.40; H, 9.05; N, 6.86. MS (LIFDI, THF): *m/z* = 607.4 ([M$^+$]).

**LSi(Se)-P-$^{Me}$cAAC (2):** Compound **A** (200 mg, 0.35 mmol) and elemental selenium (27 mg, 0.35 mmol) were placed in a Schlenk flask and toluene (20 ml) was added at room temperature. Then the reaction mixture was stirred for 12 h at room temperature. During that time the color of the reaction mixture changes from orange red to yellow. After filtration, the filtrate was reduced to 2 mL and stored at room temperature. After 1 day, compound **2** was obtained as yellow crystals (170 mg, 75%). Mp: 238 °C. $^{1}H$ NMR (500 MHz, $C_6D_6$, 298 K, ppm): $\delta$ = 1.00 (s, 6 H, C$H_3$), 1.24 (d, 6 H, CH$Me_2$), 1.30 (s, 18 H, t-Bu), 1.63 (d, 6 H, CH$Me_2$), 1.84 (s, 2 H, C$H_2$), 2.40 (s, 6 H, C$H_3$), 3.02 (sept, 2 H, C$H$Me$_2$), 6.58−7.10 (m, 8 H, $C_6H_3$ and $C_6H_5$). $^{13}C\{^{1}H\}$ NMR (125.8 MHz, $C_6D_6$, 298 K, ppm): $\delta$ = 24.21, 27.67, 27.74, 29.12, 29.43, 31.44, 33.21, 52.15, 52.24, 55.12, 56.52, 70.29, 125.17,127.63, 127.91, 128.10, 128.26, 128.30, 129.30, 129.81, 131.67, 134.46, 147.73, 171.72, 221.21 (d, $^{1}J_{CP}$ = 78 Hz). $^{31}P\{^{1}H\}$ NMR (202 MHz, 298 K,

$C_6D_6$) $\delta$ +9.37; $^{29}$Si{$^1$H} NMR (99 MHz, 298 K, $C_6D_6$, ppm) δ= +16.96 ppm ($^1J_{SiP}$ = 117 Hz). $^{77}$Se{$^1$H} NMR (95 MHz, 298 K, $C_6D_6$, ppm) δ= -286.13 ppm ($^2J_{SeP}$ = 18 Hz). Anal. Calcd (%) for $C_{35}H_{54}N_3PSeSi$ (Mw = 654.86): C, 64.19; H, 8.31; N, 6.42. Found: C, 62.65; H, 7.94; N, 6.34. MS (LIFDI, THF): *m/z* = 655.3 ([$M^+$]).

**LSi(Te)-P-$^{Me}$cAAC (3):** Compound **A** (200 mg, 0.35 mmol) and elemental tellurium (49 mg, 0.38 mmol) were placed in a Schlenk flask and toluene (20 ml) was added at room temperature. Then the reaction mixture was stirred for 12 h at 60 °C. During that time the color of the reaction mixture changes from orange red to yellow. After filtration, the filtrate was reduced to 2 mL and stored at room temperature. After 1 day, compound **3** was obtained as yellow crystals (193 mg, 79%). Mp: 232 °C. $^1$H NMR (500 MHz, $C_6D_6$, 298 K, ppm): $\delta$ = 0.97 (s, 6 H, C$H_3$), 1.22 (d, 6 H, CH$Me_2$), 1.32 (s, 18 H, t-Bu), 1.60 (d, 6 H, CH$Me_2$), 1.74 (s, 2 H, C$H_2$), 2.38 (s, 6 H, C$H_3$), 2.98 (sept, 2 H, C*H*Me$_2$), 6.57−7.08 (m, 8 H, $C_6H_3$ and $C_6H_5$). $^{13}$C{$^1$H} NMR (125.8 MHz, $C_6D_6$, 298 K, ppm): $\delta$ = 24.18, 27.70, 27.77, 29.10, 29.47, 31.57, 34.08, 52.76, 52.85, 55.49, 57.04, 70.61, 125.20,127.56, 127.91, 128.10, 128.29, 129.39, 129.71, 129.87, 132.01, 134.25, 147.63, 170.91, 221.00 (d, $^1J_{CP}$ = 78 Hz). $^{31}$P{$^1$H} NMR (202 MHz, 298 K, $C_6D_6$) $\delta$ +21.67; $^{29}$Si{$^1$H} NMR (99 MHz, 298 K, $C_6D_6$, ppm) δ= -10.94 ppm ($^1J_{SiP}$ = 128 Hz). $^{125}$Te{$^1$H} NMR (158 MHz, 298 K, $C_6D_6$, ppm) δ= -835.45 ppm ($^2J_{TeP}$ = 38 Hz). Anal. Calcd (%) for $C_{35}H_{54}N_3PSiTe$ (Mw = 703.50): C, 59.76; H, 7.74; N, 5.97. Found: C, 59.32; H, 7.11; N, 5.94. MS (LIFDI, THF): *m/z* = 705.4 ([$M^+$]).

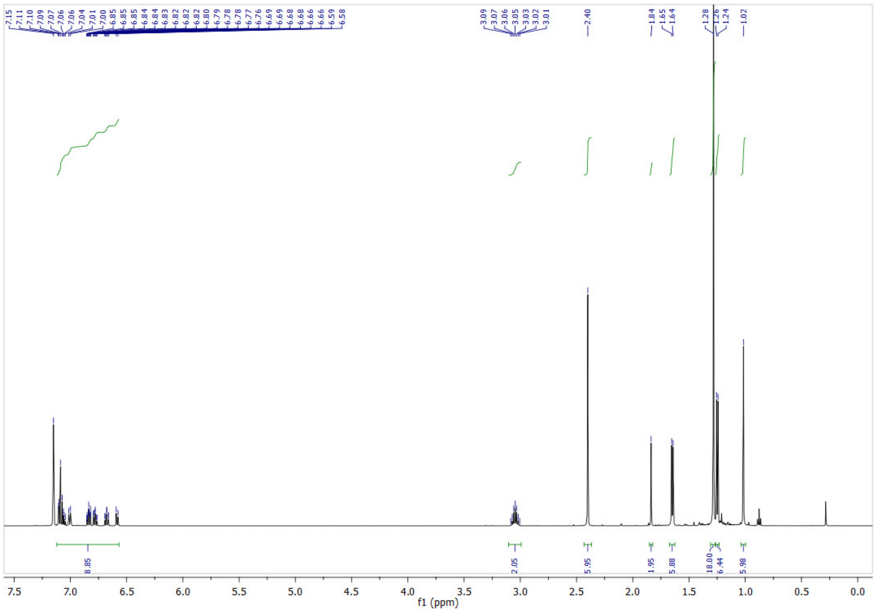


**Fig. 1**: $^{1}$H NMR of compound **1**.

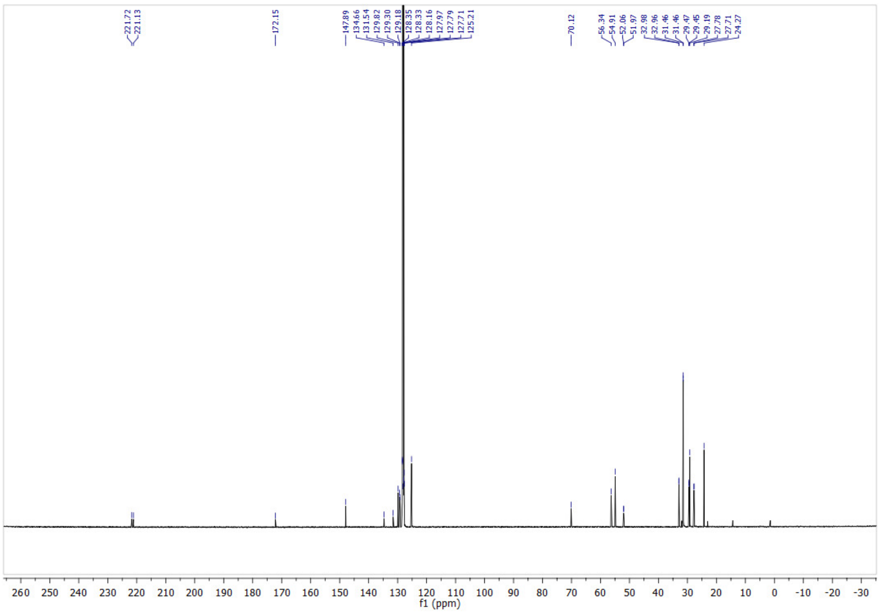


**Fig. 2**: $^{13}$C NMR of compound **1**.

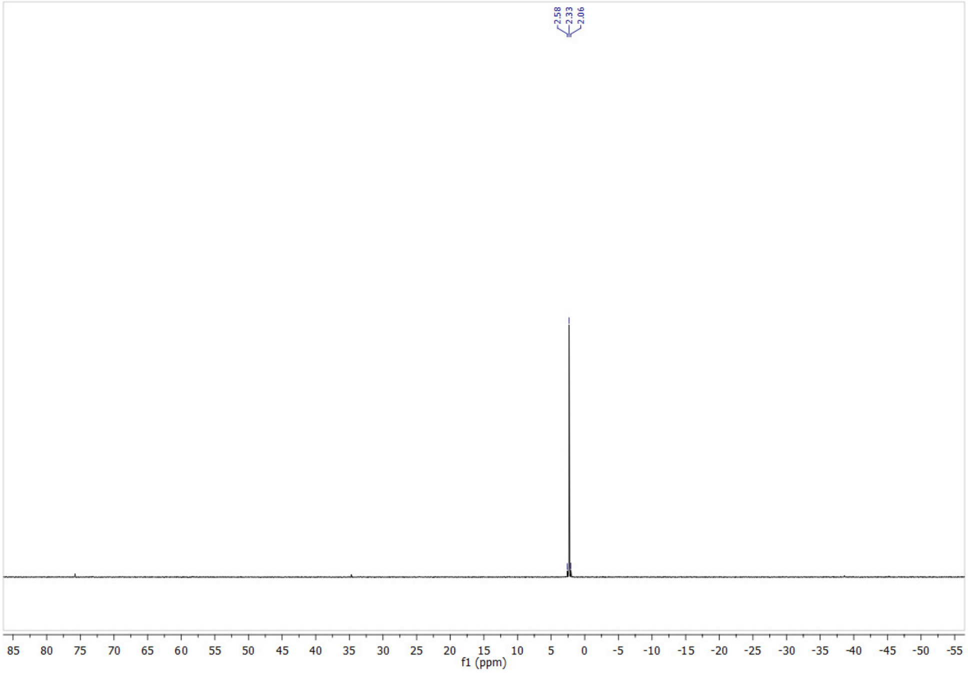


**Fig. 3**: $^{31}$P NMR of compound **1**.

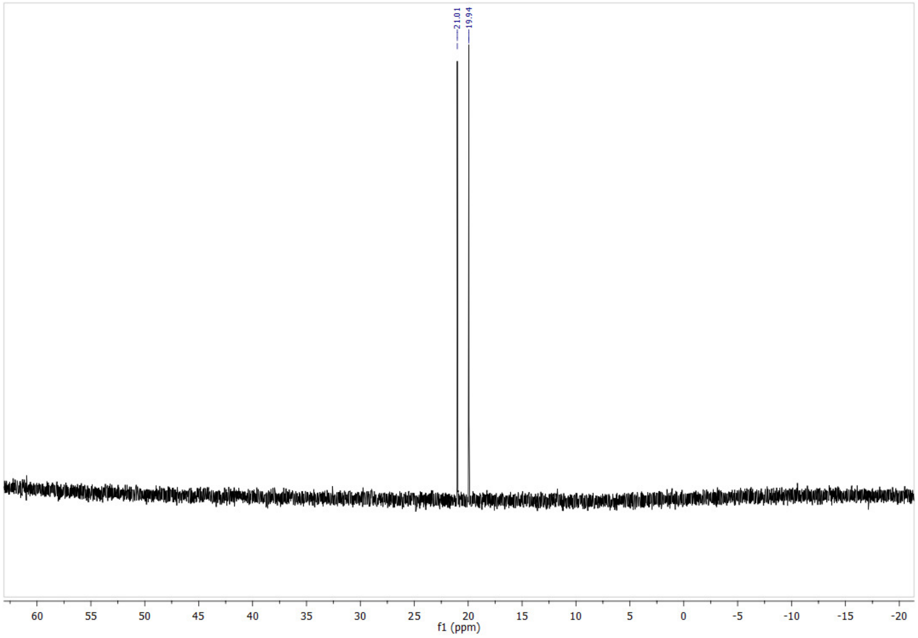


**Fig. 4**: $^{29}$Si NMR of compound **1**.

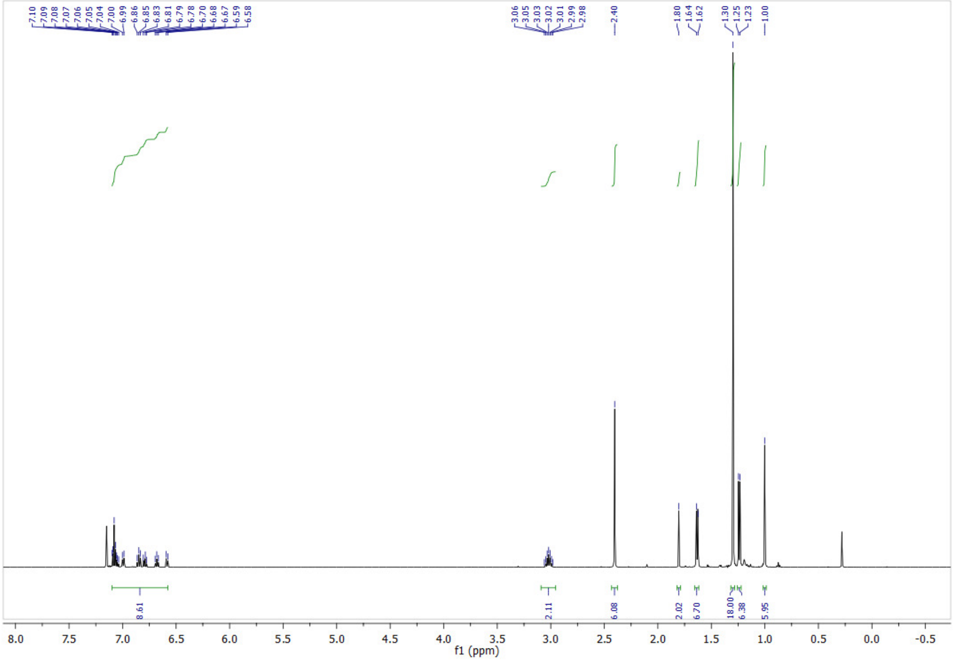


**Fig. 5**: $^{1}$H NMR of compound **2**.

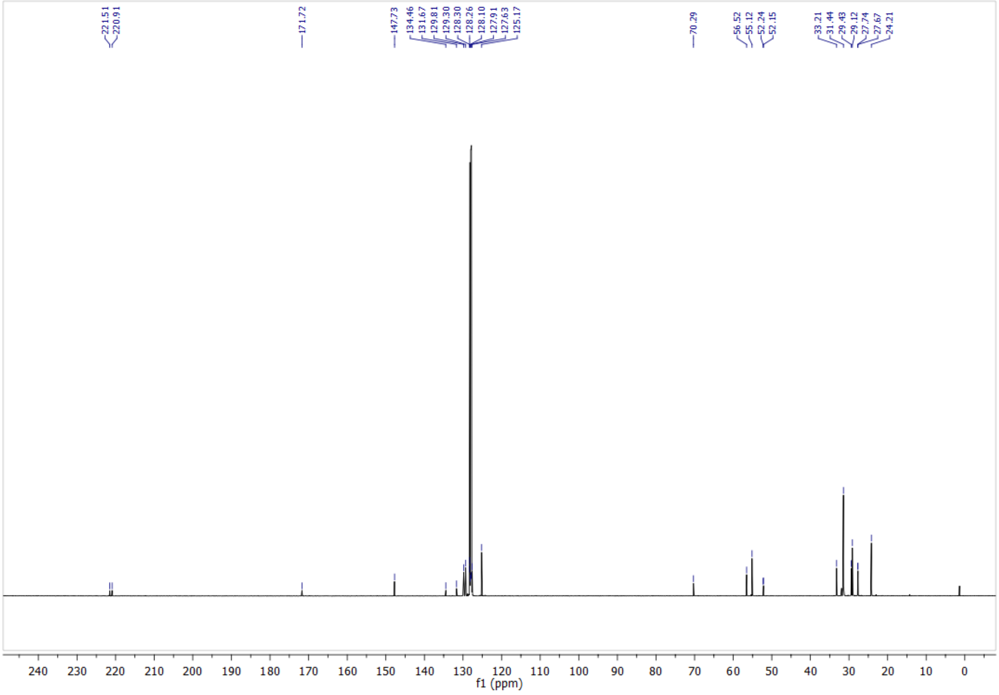

**Fig. 6**: $^{13}C$ NMR of compound **2**.

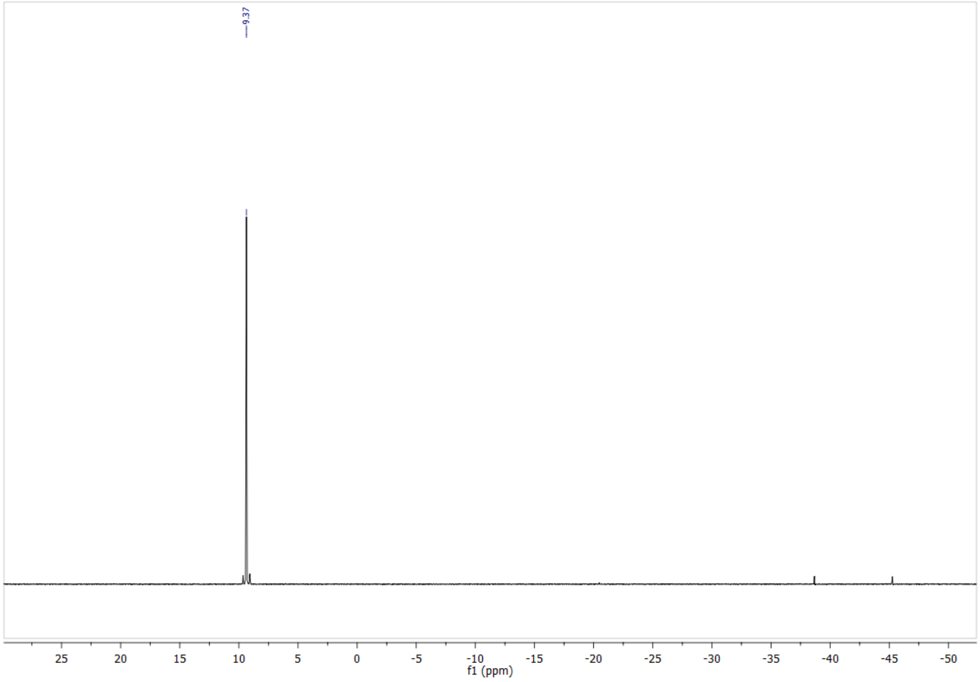


**Fig. 7**: $^{31}P$ NMR of compound **2**.

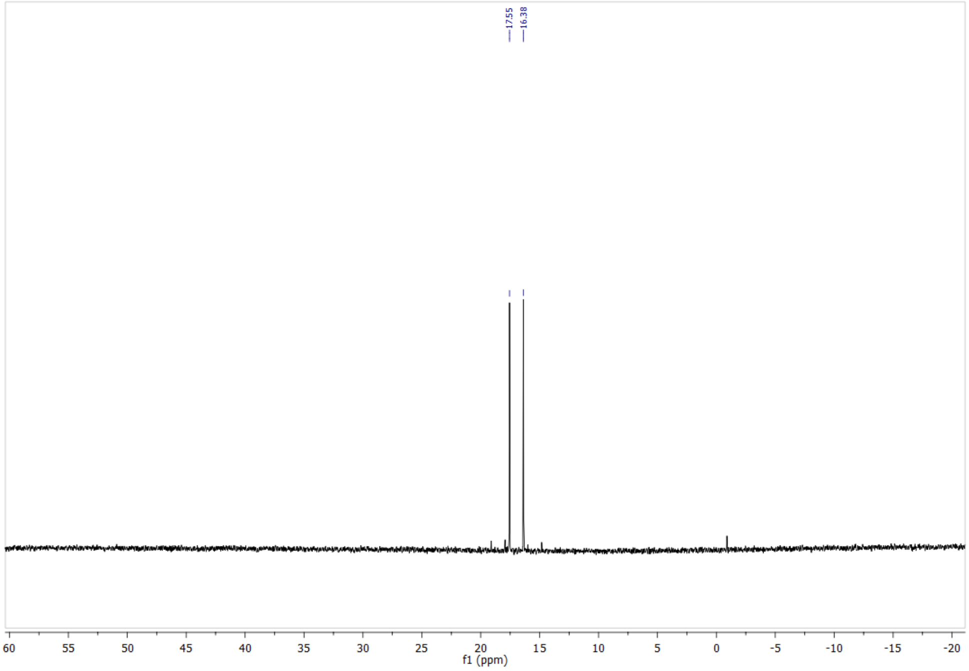


**Fig. 8**: $^{29}$Si NMR of compound **2**.

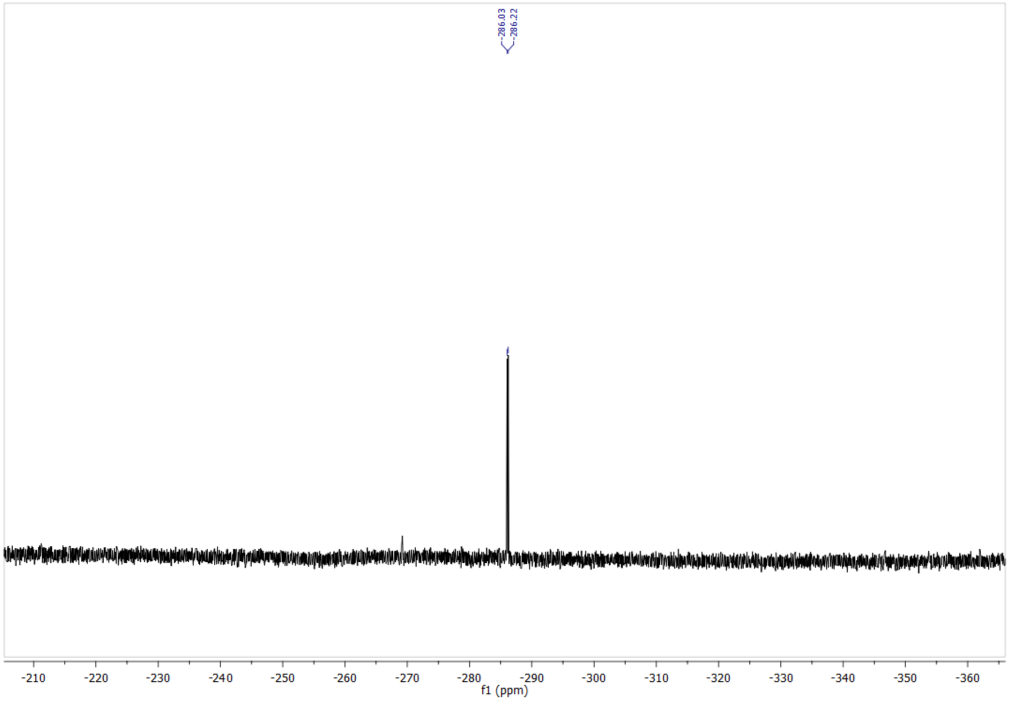

**Fig. 9**: $^{77}$Se NMR of compound **2**.

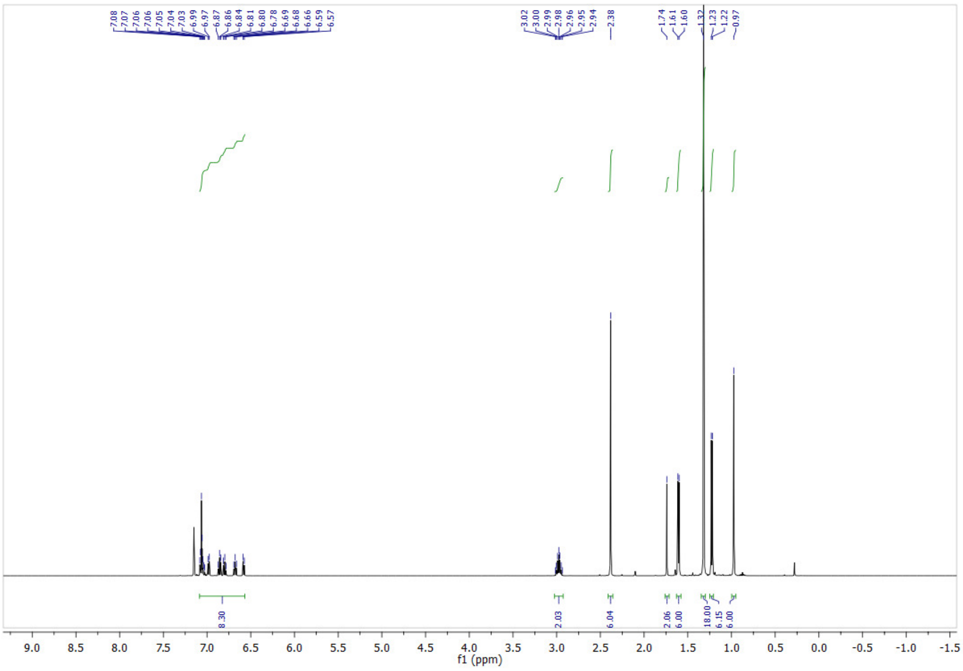


**Fig. 10**: $^{1}$H NMR of compound **3**.

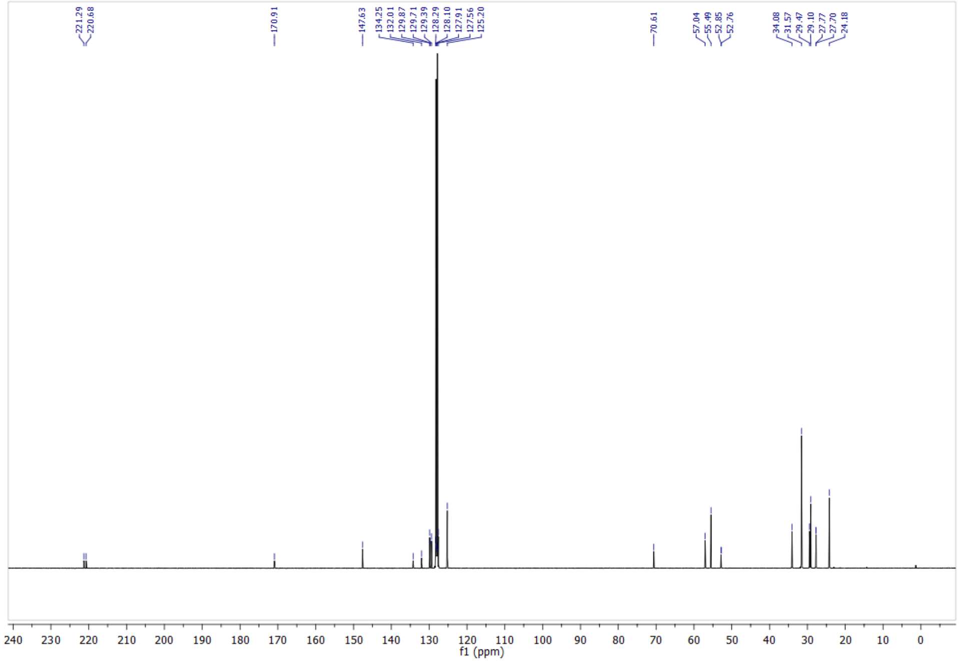


**Fig. 11**: $^{13}$C NMR of compound **3**.

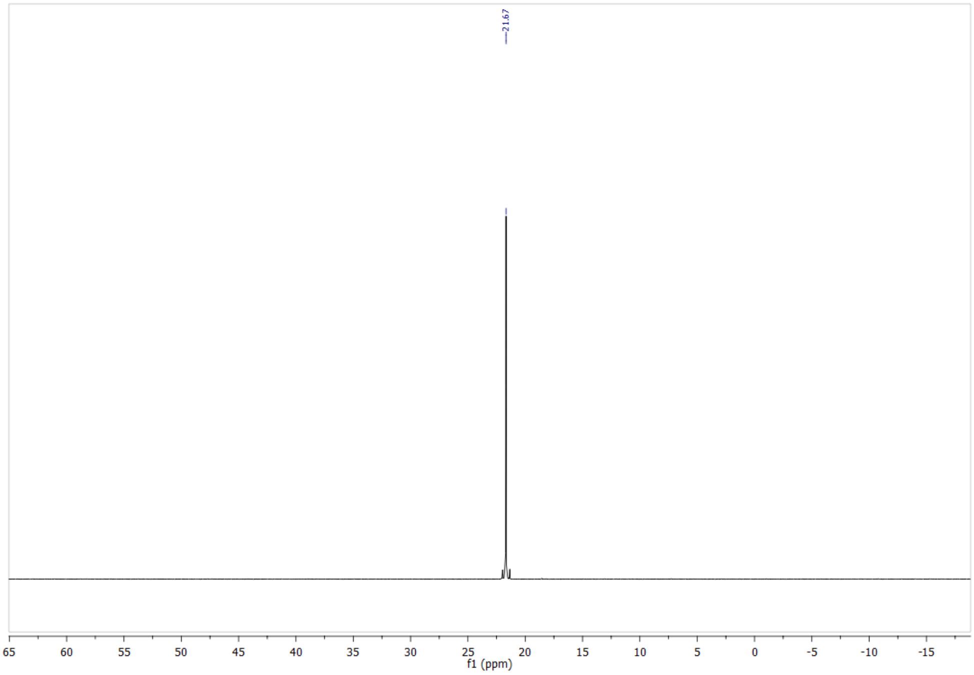

**Fig. 12**: $^{31}$P NMR of compound **3**.

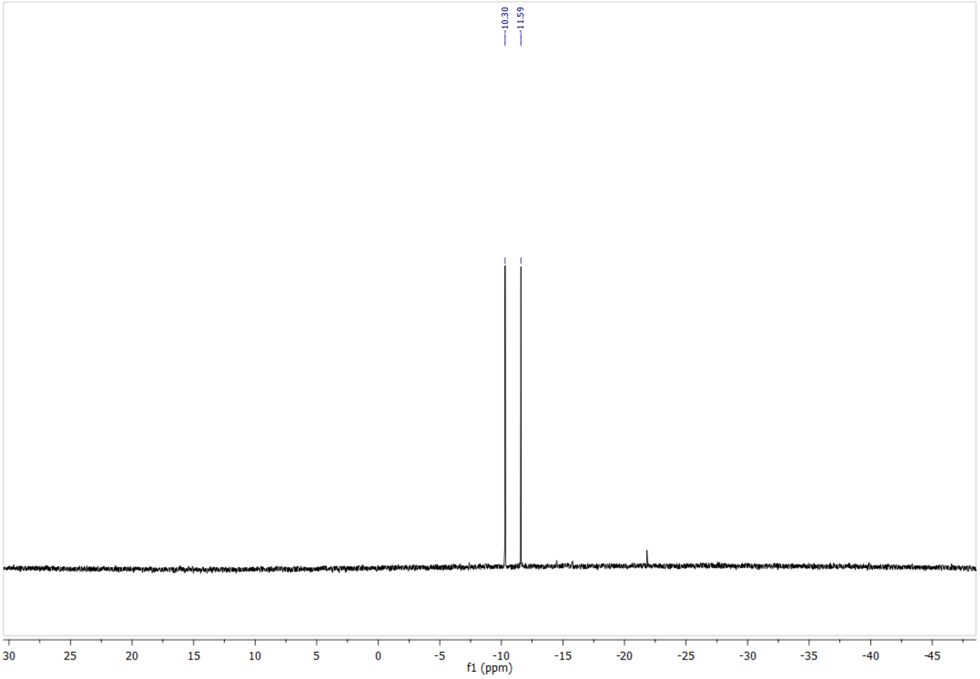


**Fig. 13**: $^{29}$Si NMR of compound **3**.

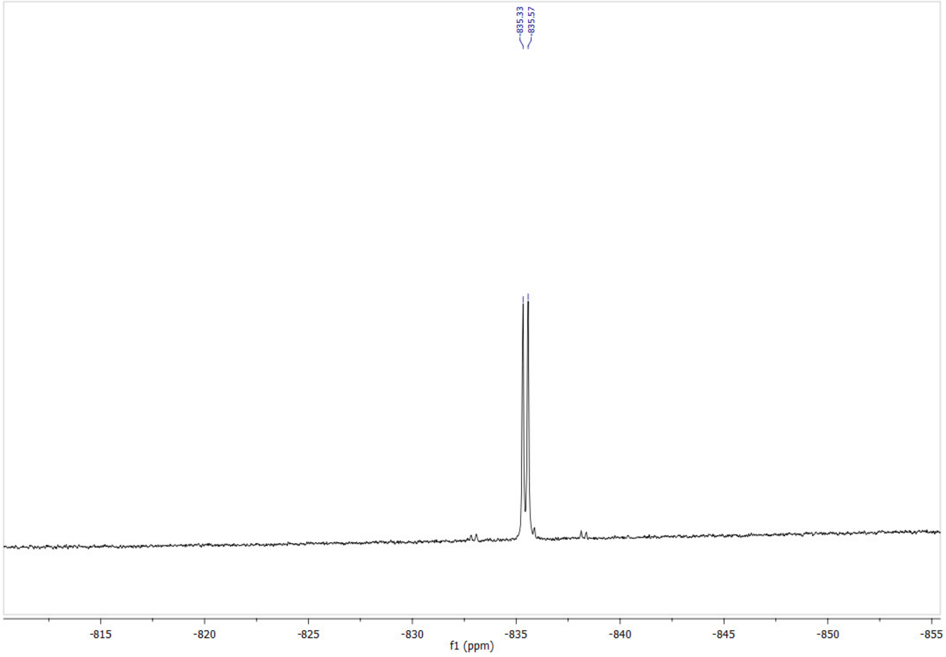


**Fig. 14**: $^{125}$Te NMR of compound **3**.

**Reaction of LSi(Se)-P-*^Me^*cAAC (2) with L'Al (L'= HC{(CMe)(2,6-*i*-Pr$_2$C$_6$H$_3$N)}$_2$)**: LSi(Se)-P-$^{Me}$cAAC **(2)** (20 mg, 0.031 mmol) and L'Al (14 mg, 0.031 mmol) were placed in a j Young NMR tube and 0.6 mL C$_6$D$_6$ was added. Then the NMR tube was heated for 6 h at 60 °C. During that time the colorless precipitate of (L'AlSe)$_2$ was deposited and LSi(Se)-P-$^{Me}$cAAC (**2**) was converted to LSi-P-$^{Me}$cAAC (**A**).

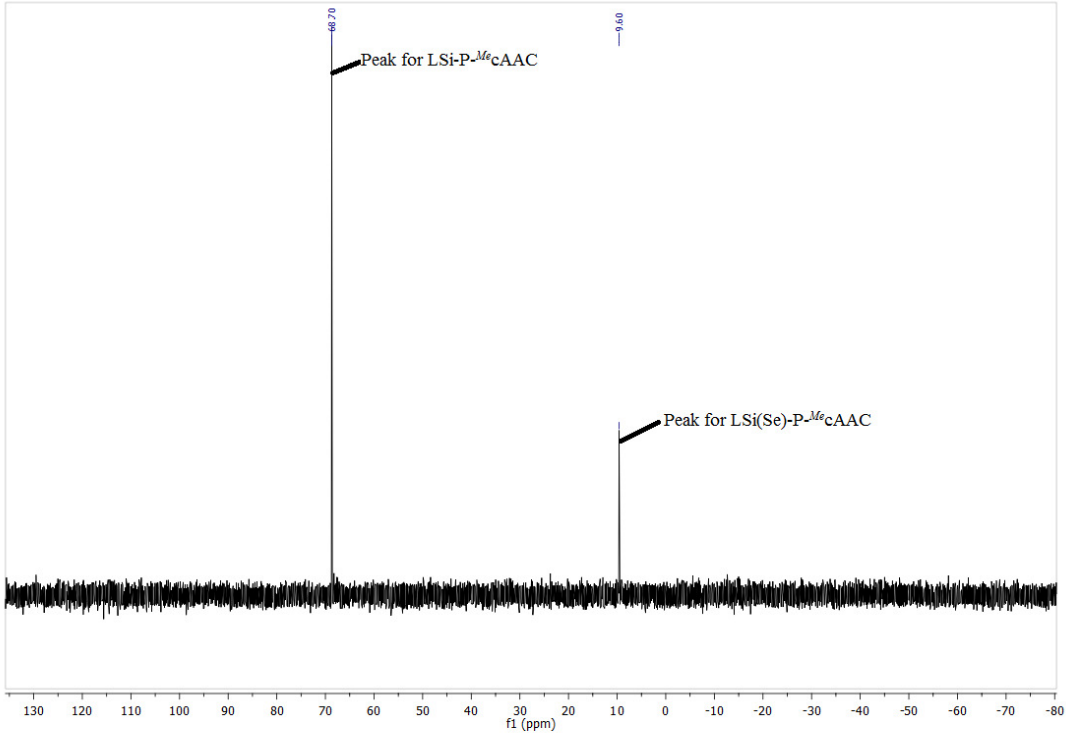


**Fig. 15**: $^{31}$P NMR of reaction of LSi(Se)-P-$^{Me}$cAAC (**2**) with L'Al.

**Reaction of LSi(Te)-P-*$^{Me}$*cAAC (3) with L'Al**: LSi(Te)-P-*$^{Me}$*cAAC **(2)** (20 mg, 0.028 mmol) and L'Al (13 mg, 0.028 mmol) were placed in a j Young NMR tube and 0.6 mL $C_6D_6$ was added. Then the NMR tube was heated for 6 h at 60 °C. During that time the colorless precipitate of $(L'AlTe)_2$ was deposited and LSi(Te)-P-*$^{Me}$*cAAC (**3**) was converted to LSi-P-*$^{Me}$*cAAC (**A**).

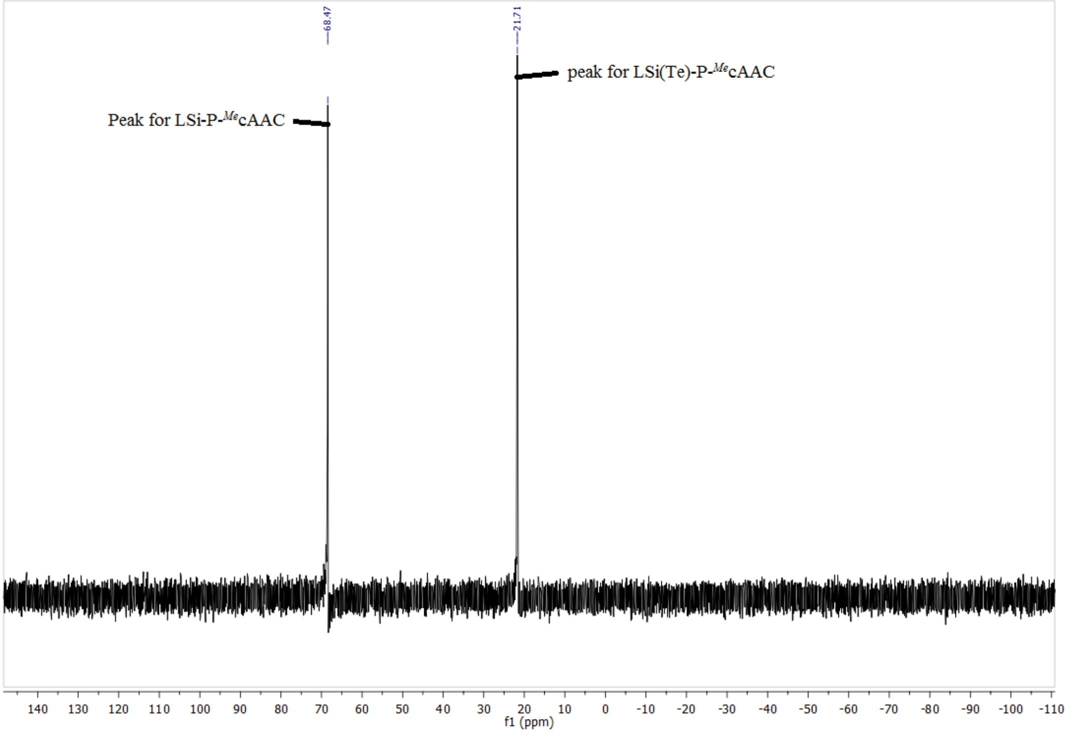


**Fig. 16**: $^{31}$P NMR of reaction of LSi(Te)-P-*$^{Me}$*cAAC (**3**) with L'Al.

**S2. Theoretical analysis**

Geometry optimizations of all the molecules were performed at the BP86[2]/def2-SVP[3] level of theory, using Gaussian 09 programme[4] package. Single point calculations were carried out at the M06[5]/def2-TZVPP[3] level of theory using the optimized geometry at the BP86[2]/def2-SVP[3] level of theory. Natural Bond Orbital analysis (NBO)[6] was done at the M06/def2-TZVPP//BP86/def2-SVP level of theory. The analysis of the electrostatic potential (ESP) on the molecular van der Waals surface were done at the M06/def2-TZVPP//BP86/def2-SVP level of theory using the Multiwfn programme.[7]

Energy Decomposition Analysis combined with Natural Orbital for Chemical Valence (EDA-NOCV) analysis was carried out at the BP86[2]/TZ2P[3] level of theory using the ADF2016.107 program.[8] Basis sets from ZORA[9] (Zeroth Order Regular Approximation) were taken for calculation. The core electrons were treated with the frozen core approximations. Scalar relativistic effects were also incorporated. According to EDA analysis[10] the interaction energy $\Delta E_{int}$ between two fragments in the frozen geometry of the molecule can be divided into three energy components as:

$$\Delta E_{int} = \Delta E_{elstat} + \Delta E_{Pauli} + \Delta E_{orb}$$

$\Delta E_{elstat}$ gives the energy due to electrostatic interaction between the fragments, $\Delta E_{Pauli}$ represents the energy due to the repulsion between electrons with same spin in the fragments, and $\Delta E_{orb}$ is the energy due to overlap of the orbitals in the fragments. The extension of EDA with NOCV[11] scheme provides information about the different types of orbital interactions.

The $\Delta E_{int}$ is related to the preparatory energy (energy required to excite the fragments from the ground geometrical as well as the electronic state to the geometry and electronic state in the molecule) and dissociation energy as follows:

$-D_e = \Delta E_{int} + \Delta E_{prep}$

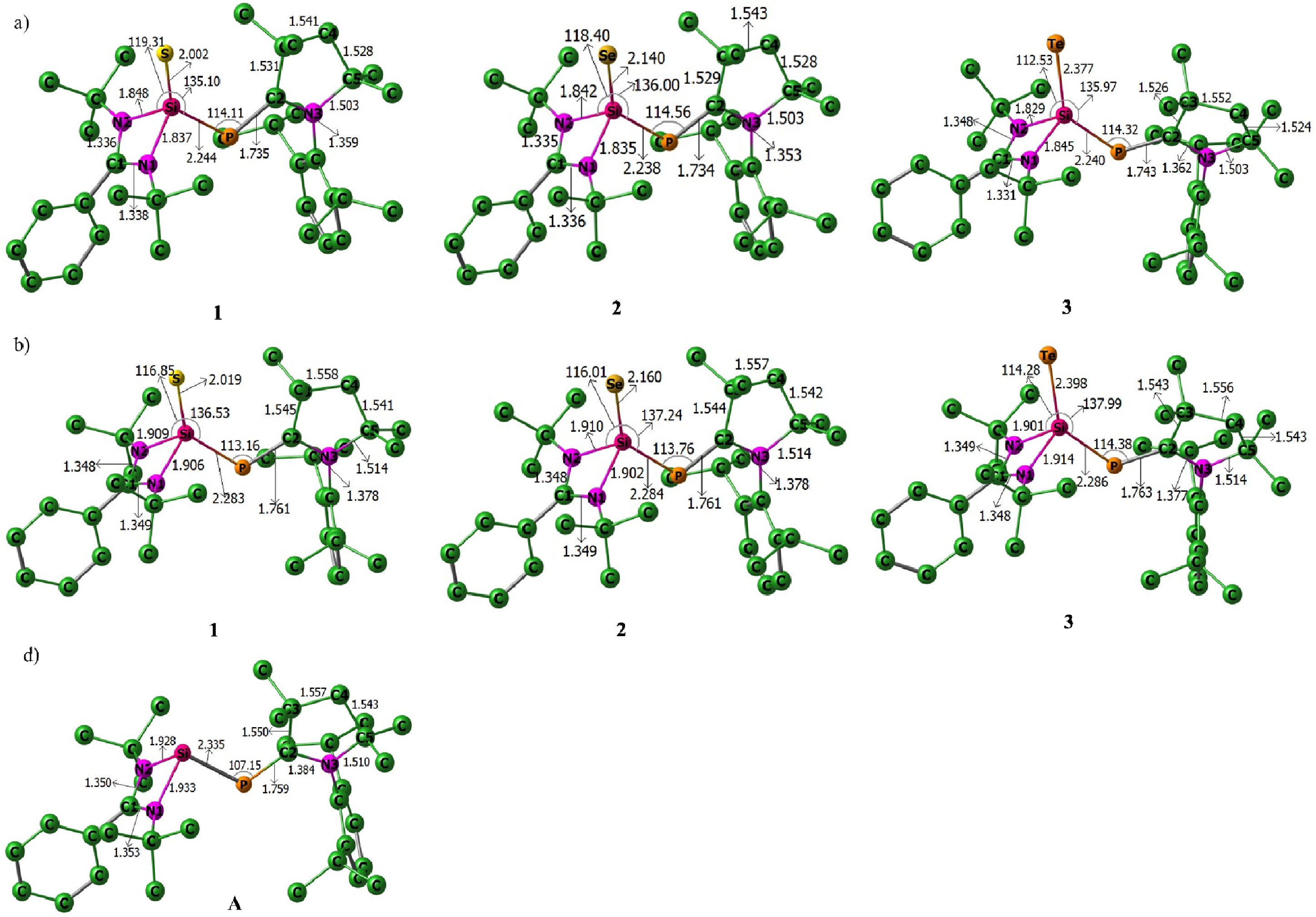


**Figure S2.1**: a) Experimental geometry of **1**, **2** and **3** and b) optimized (BP86/def2-SVP) geometry of **1**, **2** and **3**, c) optimized geometry of parent molecule **A**.

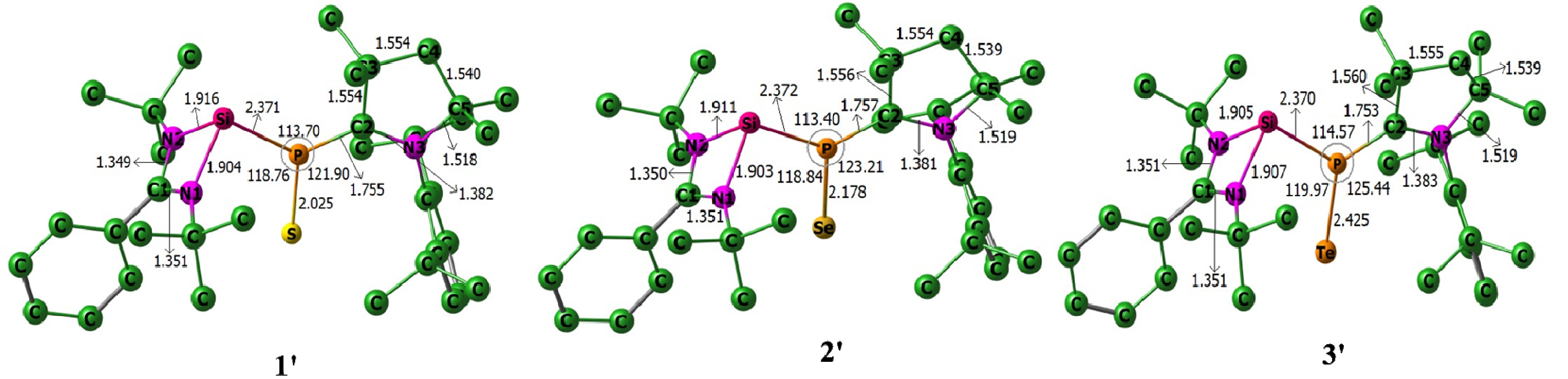


**1′** **2′** **3′**

**Figure S2.2**: Optimized (BP86/def2-SVP) geometry of hypothetical chalcogen bonded phosphorous phosphinidenes **1′**, **2′** and **3′**.

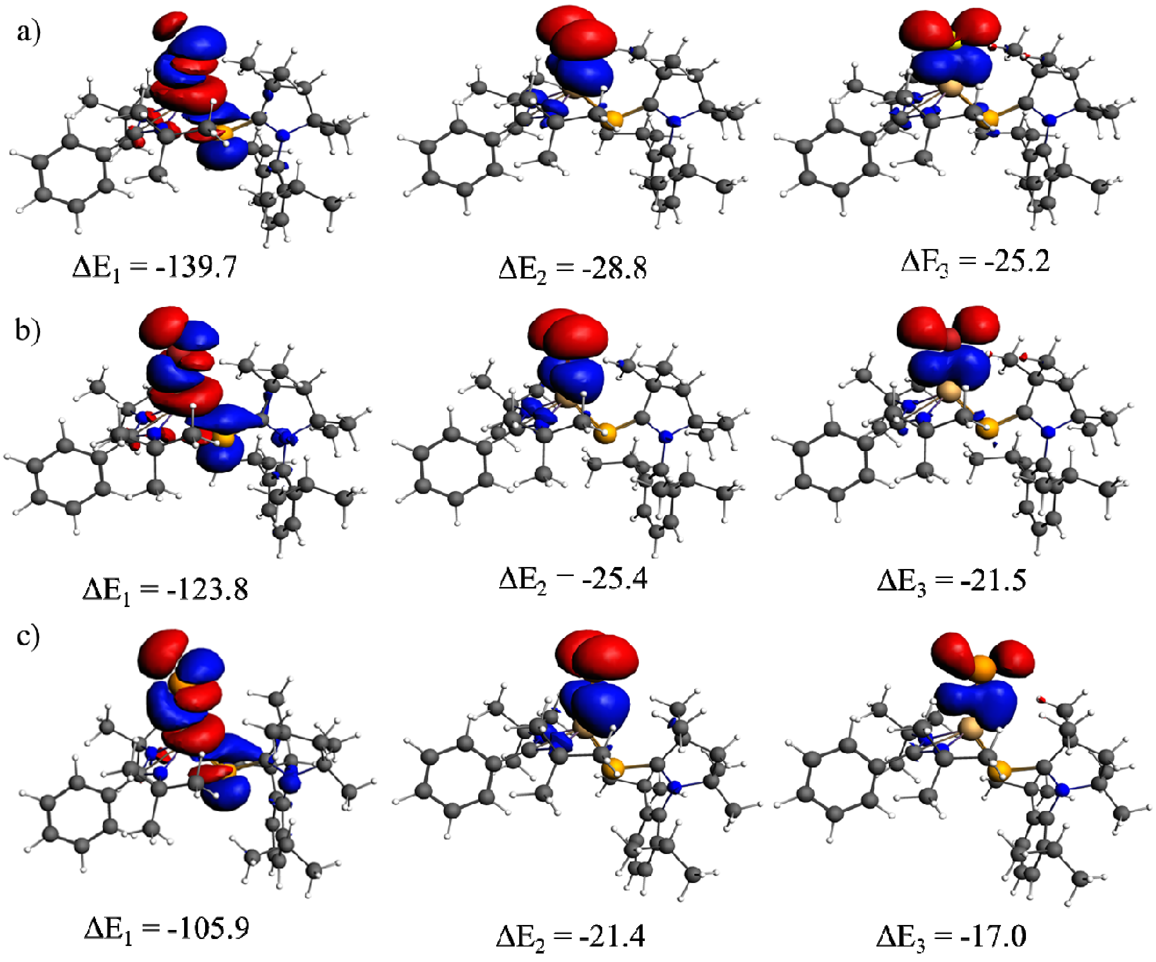


**Figure S2.3**: Plots of deformation densities (BP86/TZ2P) for the interaction of [LSi-P-${}^{Me}$cAAC]$^+$ with E$^-$ in a) **1** b) **2**, and c) **3** where L represents amidinate ligand [(Ph)C(N*t*-Bu)$_2$]. The direction of charge flow is from red to blue. The isosurface value for the plot is 0.001. The associated energy (ΔE) is given in kcal/mol.

**Table S2.1**: ESP values in kcal/mol (M06/def2-TZVPP//BP86/def2-SVP) at local surfaces corresponding to some important atoms in **A**, **1**, **2** and **3**.

| Molecule | Atom | ESP |
|---|---|---|
| **A** | Si | -26.0 |
| | P | -30.3 |
| **1** | S | -35.5 |
| | P | -24.2 |
| **2** | Se | -31.6 |
| | P | -23.4 |
| **3** | Te | -25.5 |
| | P | -23.2 |

**Table S2.2**: Occupancy at the lone Pairs on E (S/Se/Te) and P atoms in **1-3** by natural bond orbital analysis at M06/def2-TZVPP//BP86/def2-SVP level of theory.

| Atom | Lone pair occupancy | | | |
|---|---|---|---|---|
| | **A** | **1** | **2** | **3** |
| E | – | 1.959 | 1.957 | 1.959 |
| | | 1.804 | 1.801 | 1.802 |
| P | 1.943 | 1.908 | 1.903 | 1.897 |
| Si | 1.925 | – | – | – |

**Table S2.3**: Second Order Perturbation Analysis at the M06/def2-TZVPP//BP86/def2-SVP level of theory.

| Compound | Donor orbital | Acceptor orbital | Energy (kcal/mol) |
|---|---|---|---|
| **A** | LP(1)P | BD*(1)C2–C3 | 16.0 |
| **1** | LP (1)P | BD*(1)C2–C3 | 15.3 |
| | LP (1)P | BD* (1)S–Si | 8.7 |
| **2** | LP (1)P | BD*(1)C2–C3 | 15.2 |
| | LP (1)P | BD* (1)Se–Si | 9.2 |
| **3** | LP (1)P | BD*(1)C2–C3 | 15.1 |
| | LP (1)P | BD* (1)Te–Si | 10.7 |

**Table S2.4**: Reaction energy for the formation of chalcogen bonded silicon phosphinidenes **1**, **2** and **3** as well as hypothetical chalcogen bonded phosphorous phosphinidenes **1′**, **2′** and **3′** in terms of enthalpy (ΔE) and Gibb's free energy (ΔG). Energy is expressed in kcal/mol.

| Reaction | ΔE | ΔG | Reaction | ΔE | ΔG |
|---|---|---|---|---|---|
| **A** + S → **1** | -107.3 | -107.5 | **A** + S → **1'** | -77.0 | -76.7 |
| **A** + Se → **2** | -88.2 | -89.6 | **A** + Se → **2'** | -59.4 | -60.2 |
| **A** + Te → **3** | -71.2 | -72.6 | **A** + Te → **3'** | -46.8 | -49.5 |

**Table S2.5**: EDA-NOCV results of different bonding representations for Si–E bond in **1**, **2**, and **3**. Here**,** L represents amidinate ligand [(Ph)C(N*t*-Bu)$_2$]. Energy is given in kcal/mol.

| Compound | Bonding representation | $\Delta E_{int}$ | $\Delta E_{Pauli}$ | $\Delta E_{elstat}$ | $\Delta E_{orb}$ |
|---|---|---|---|---|---|
| **1** | [LSi-P-$^{Me}$cAAC]$^+$↔S$^-$ | -181.9 | 277.6 | -243.6 | -215.8 |
| | [LSi-P-$^{Me}$cAAC] →S | -145.7 | 286.7 | -167.1 | -265.2 |
| **2** | [LSi-P-$^{Me}$cAAC]$^+$↔Se$^-$ | -167.7 | 255.0 | -233.6 | -189.1 |
| | [LSi-P-$^{Me}$cAAC] →Se | -128.0 | 260.1 | -173.5 | -214.7 |
| **3** | [LSi-P-$^{Me}$cAAC]$^+$ ↔Te$^-$ | -149.2 | 217.4 | -207.0 | -159.6 |
| | [LSi-P-$^{Me}$cAAC] →Te | -104.8 | 223.4 | -163.3 | -164.9 |

**Table S2.6.** EDA-NOCV results (BP86/TZ2P) for the charge separated electron sharing possibility ([LSi-P-$^{Me}$cAAC]$^{+}$↔E$^{-}$), in **1**, **2**, and **3**, where L represents amidinate ligand [(Ph)C(N*t*-Bu)$_2$]. Energies are in kcal/mol.

| Molecule | **1** | **2** | **3** |
|---|---|---|---|
| **$\Delta E_{int}$** | -181.9 | -167.7 | -149.2 |
| **$\Delta E_{Pauli}$** | 277.6 | 255.0 | 217.4 |
| **$\Delta E_{elstat}$**$^a$ | -243.6 (53.0%) | -233.6 (55.3%) | -207.0 (56.5%) |
| **$\Delta E_{orb}$**$^a$ | -215.8 (47.0%) | -189.1 (44.7%) | -159.6 (43.5%) |
| **$\Delta E_1$**$^b$ | -139.7 (64.7%) | -123.8 (65.5 %) | -105.9 (66.4%) |
| **$\Delta E_2$**$^b$ | -28.8 (13.4%) | -25.4 (13.4%) | -21.4 (13.4 %) |
| **$\Delta E_3$**$^b$ | -25.2 (11.7%) | -21.5 (11.4 %) | -17.0 (10.6%) |
| **$\Delta E_{rest}$**$^{b,c}$ | -22.1 (10.2%) | -18.4 (9.7%) | -15.3 (9.6%) |
| **$\Delta E_{prep}$**$^d$ | 76.0 | 77.2 | 77.9 |
| **-$D_e$**$^d$ | -105.9 | -90.5 | -71.3 |

$^a$Values in parentheses give the percentage contribution to the total attractive interactions, $\Delta E_{orb}$ + $\Delta E_{elstat}$. $^b$Values in parentheses give the percentage contribution to the orbital interactions, $\Delta E_{orb}$. $^c\Delta E_{rest}$ = $\Delta E_{orb}$ - ($\Delta E_1$+ $\Delta E_2$+ $\Delta E_3$). $^d\Delta E_{prep}$ and $D_e$ represent the preparatory and dissociation energy respectively.

**Table S2.7**: Cartesian coordinates, electronic energy $E^{el}_{M06}$ (M06/def2-TZVPP//BP86/def2-SVP), zero-point energy $ZPE_{BP86}$ (BP86/def2-SVP) and total energy E ($E^{el}_{M06}$ + $ZPE_{BP86}$) of molecules mentioned in the paper using Gaussian09 program package. The energies are given in a.u.

**1**

$E^{el}_{M06}$ = -2559.0136988

$ZPE_{BP86}$ = 0.800837

E ($E^{el}_{M06}$ + $ZPE_{BP86}$) = -2558.2128618

| | | | |
|---|---|---|---|
| 16 | -1.689386000 | 3.460197000 | -0.966820000 |
| 15 | 0.429819000 | 0.190174000 | -0.072147000 |
| 14 | -1.376980000 | 1.525035000 | -0.481490000 |
| 7 | -2.554101000 | 0.240007000 | -1.260079000 |
| 6 | -2.670830000 | -0.426898000 | -2.578033000 |
| 6 | -2.328881000 | -1.930071000 | -2.471054000 |
| 1 | -2.305045000 | -2.389359000 | -3.481414000 |
| 1 | -3.080599000 | -2.479091000 | -1.869400000 |
| 1 | -1.333478000 | -2.066608000 | -2.001281000 |
| 6 | -4.087651000 | -0.231276000 | -3.165594000 |
| 1 | -4.123832000 | -0.630881000 | -4.200482000 |
| 1 | -4.346875000 | 0.846552000 | -3.201032000 |
| 1 | -4.858125000 | -0.763239000 | -2.573471000 |
| 6 | -1.648316000 | 0.273019000 | -3.498902000 |
| 1 | -1.690812000 | -0.162495000 | -4.517835000 |
| 1 | -0.617468000 | 0.147293000 | -3.107722000 |
| 1 | -1.860459000 | 1.359542000 | -3.565524000 |
| 6 | -3.164536000 | 0.030421000 | -0.076782000 |
| 7 | -2.557542000 | 0.816267000 | 0.836494000 |
| 6 | -2.983244000 | 1.285841000 | 2.178152000 |
| 6 | -3.290602000 | 0.110592000 | 3.130237000 |
| 1 | -3.493283000 | 0.504547000 | 4.147815000 |
| 1 | -2.424420000 | -0.579459000 | 3.194940000 |
| 1 | -4.179180000 | -0.468786000 | 2.813148000 |
| 6 | -4.213606000 | 2.212823000 | 2.029992000 |
| 1 | -4.471613000 | 2.674261000 | 3.006334000 |
| 1 | -5.101696000 | 1.648554000 | 1.678673000 |
| 1 | -3.992059000 | 3.018805000 | 1.300606000 |
| 6 | -1.800744000 | 2.099953000 | 2.746577000 |
| 1 | -2.051719000 | 2.475369000 | 3.759555000 |
| 1 | -1.577733000 | 2.969893000 | 2.095385000 |
| 1 | -0.888595000 | 1.473383000 | 2.818329000 |
| 6 | -4.294956000 | -0.915042000 | 0.186474000 |
| 6 | -4.024086000 | -2.210074000 | 0.682929000 |
| 1 | -2.981698000 | -2.511845000 | 0.868366000 |
| 6 | -5.077551000 | -3.101204000 | 0.944583000 |
| 1 | -4.855776000 | -4.108933000 | 1.329951000 |
| 6 | -6.409174000 | -2.708367000 | 0.719710000 |
| 1 | -7.234252000 | -3.407455000 | 0.928805000 |
| 6 | -6.684130000 | -1.420368000 | 0.227907000 |
| 1 | -7.724972000 | -1.106460000 | 0.050975000 |
| 6 | -5.633497000 | -0.526616000 | -0.042040000 |
| 1 | -5.850118000 | 0.480128000 | -0.431114000 |
| 6 | 1.967962000 | 1.013671000 | -0.312362000 |
| 6 | 2.330555000 | 2.422671000 | -0.833142000 |
| 6 | 3.879609000 | 2.342903000 | -0.981625000 |
| 1 | 4.131245000 | 2.102651000 | -2.036202000 |
| 1 | 4.370270000 | 3.307219000 | -0.738849000 |
| 6 | 4.379235000 | 1.198434000 | -0.078044000 |
| 7 | 3.132208000 | 0.342675000 | -0.006180000 |
| 6 | 1.701839000 | 2.706101000 | -2.215533000 |
| 1 | 2.119548000 | 3.653401000 | -2.620674000 |
| 1 | 0.602589000 | 2.832655000 | -2.146970000 |
| 1 | 1.936268000 | 1.893888000 | -2.935123000 |
| 6 | 1.894408000 | 3.519662000 | 0.169040000 |
| 1 | 2.275814000 | 4.505847000 | -0.171789000 |
| 1 | 2.285580000 | 3.335151000 | 1.190307000 |
| 1 | 0.788115000 | 3.588159000 | 0.214299000 |
| 6 | 5.570579000 | 0.449056000 | -0.694256000 |
| 1 | 5.862660000 | -0.431061000 | -0.085735000 |
| 1 | 6.442466000 | 1.133690000 | -0.737865000 |
| 1 | 5.358678000 | 0.110087000 | -1.725560000 |
| 6 | 4.797094000 | 1.704315000 | 1.319512000 |
| 1 | 5.109070000 | 0.865658000 | 1.971659000 |
| 1 | 3.981399000 | 2.258195000 | 1.822883000 |
| 1 | 5.663219000 | 2.390763000 | 1.219007000 |
| 6 | 3.177765000 | -1.054093000 | 0.372185000 |
| 6 | 3.338087000 | -2.035854000 | -0.656659000 |

| | | | |
|---|---|---|---|
| 6 | 3.476327000 | -3.387078000 | -0.279257000 |
| 1 | 3.608325000 | -4.152171000 | -1.060823000 |
| 6 | 3.441044000 | -3.777532000 | 1.064904000 |
| 1 | 3.561107000 | -4.838196000 | 1.338086000 |
| 6 | 3.226746000 | -2.814256000 | 2.056802000 |
| 1 | 3.160064000 | -3.130581000 | 3.110109000 |
| 6 | 3.076136000 | -1.446916000 | 1.741392000 |
| 6 | 3.294301000 | -1.700962000 | -2.150626000 |
| 1 | 3.304547000 | -0.595797000 | -2.245654000 |
| 6 | 1.971457000 | -2.197139000 | -2.777021000 |
| 1 | 1.899584000 | -1.880746000 | -3.839318000 |
| 1 | 1.099484000 | -1.785558000 | -2.230560000 |
| 1 | 1.906193000 | -3.306096000 | -2.750079000 |
| 6 | 4.499752000 | -2.263856000 | -2.932916000 |
| 1 | 4.477146000 | -1.909267000 | -3.984977000 |
| 1 | 4.483380000 | -3.373812000 | -2.964784000 |
| 1 | 5.469350000 | -1.959963000 | -2.488957000 |
| 6 | 2.727255000 | -0.483203000 | 2.878609000 |
| 1 | 2.686817000 | 0.539065000 | 2.453594000 |
| 6 | 3.769731000 | -0.496404000 | 4.017003000 |
| 1 | 3.525859000 | 0.277027000 | 4.775501000 |
| 1 | 4.798946000 | -0.301730000 | 3.651554000 |
| 1 | 3.786924000 | -1.474999000 | 4.542065000 |
| 6 | 1.321023000 | -0.795788000 | 3.440081000 |
| 1 | 1.035986000 | -0.046106000 | 4.208558000 |
| 1 | 1.290699000 | -1.797190000 | 3.920666000 |
| 1 | 0.564527000 | -0.771593000 | 2.629131000 |

**2**

$E^{el}_{M06}$ = -4562.3034722

$ZPE_{BP86}$ = 0.799841

E ($E^{el}_{M06}$ + $ZPE_{BP86}$) = -4561.5036312

| | | | |
|---|---|---|---|
| 34 | -1.839432000 | 3.441807000 | 0.388964000 |
| 15 | 0.533196000 | 0.058976000 | 0.154478000 |
| 14 | -1.350460000 | 1.351069000 | 0.154009000 |
| 7 | -2.479625000 | 0.353650000 | -1.019225000 |
| 6 | -2.815435000 | 0.415355000 | -2.460710000 |
| 6 | -2.934046000 | -0.990101000 | -3.089264000 |
| 1 | -3.064131000 | -0.893682000 | -4.187229000 |
| 1 | -3.802549000 | -1.554132000 | -2.697997000 |
| 1 | -2.015300000 | -1.583132000 | -2.903086000 |
| 6 | -4.127390000 | 1.213453000 | -2.653578000 |
| 1 | -4.330187000 | 1.367196000 | -3.734297000 |
| 1 | -4.047296000 | 2.204699000 | -2.162676000 |
| 1 | -4.993794000 | 0.673051000 | -2.221574000 |
| 6 | -1.657558000 | 1.175686000 | -3.141550000 |
| 1 | -1.853928000 | 1.272616000 | -4.228715000 |
| 1 | -0.697525000 | 0.637969000 | -3.003376000 |
| 1 | -1.554571000 | 2.194044000 | -2.713574000 |
| 6 | -3.037422000 | -0.300159000 | 0.018942000 |
| 7 | -2.433637000 | 0.139594000 | 1.142249000 |
| 6 | -2.672566000 | -0.136091000 | 2.579840000 |
| 6 | -2.486874000 | -1.635625000 | 2.904476000 |
| 1 | -2.557951000 | -1.792947000 | 4.000877000 |
| 1 | -1.489562000 | -1.987141000 | 2.569585000 |
| 1 | -3.262701000 | -2.264304000 | 2.424822000 |
| 6 | -4.087876000 | 0.338864000 | 2.984395000 |
| 1 | -4.225918000 | 0.232293000 | 4.080724000 |
| 1 | -4.877887000 | -0.258487000 | 2.487044000 |
| 1 | -4.225263000 | 1.405935000 | 2.715618000 |
| 6 | -1.625473000 | 0.685616000 | 3.362928000 |
| 1 | -1.751748000 | 0.515916000 | 4.451600000 |
| 1 | -1.737325000 | 1.769739000 | 3.156972000 |
| 1 | -0.595490000 | 0.386935000 | 3.078865000 |
| 6 | -4.113324000 | -1.337572000 | -0.065290000 |
| 6 | -3.762634000 | -2.702391000 | -0.172449000 |
| 1 | -2.699537000 | -2.988191000 | -0.191132000 |
| 6 | -4.762691000 | -3.684251000 | -0.260744000 |
| 1 | -4.478786000 | -4.745187000 | -0.344701000 |
| 6 | -6.120047000 | -3.315680000 | -0.243653000 |
| 1 | -6.903014000 | -4.087234000 | -0.313331000 |
| 6 | -6.474306000 | -1.959551000 | -0.136755000 |
| 1 | -7.535375000 | -1.664462000 | -0.121433000 |
| 6 | -5.477454000 | -0.972470000 | -0.047250000 |
| 1 | -5.757205000 | 0.088510000 | 0.041378000 |
| 6 | 2.016155000 | 0.965967000 | -0.129745000 |
| 6 | 2.296060000 | 2.464727000 | -0.374892000 |
| 6 | 3.800501000 | 2.468393000 | -0.777012000 |
| 1 | 3.879639000 | 2.463503000 | -1.884855000 |
| 1 | 4.324208000 | 3.376683000 | -0.415794000 |
| 6 | 4.438267000 | 1.176915000 | -0.225719000 |
| 7 | 3.216197000 | 0.288921000 | -0.120653000 |
| 6 | 1.461097000 | 3.047540000 | -1.534139000 |
| 1 | 1.827273000 | 4.070200000 | -1.769838000 |
| 1 | 0.390258000 | 3.139259000 | -1.261973000 |
| 1 | 1.557708000 | 2.429431000 | -2.450988000 |
| 6 | 2.028897000 | 3.280406000 | 0.914841000 |
| 1 | 2.337267000 | 4.336368000 | 0.759887000 |
| 1 | 2.589061000 | 2.882249000 | 1.785306000 |
| 1 | 0.946241000 | 3.278805000 | 1.158529000 |
| 6 | 5.493602000 | 0.598958000 | -1.182350000 |
| 1 | 5.880292000 | -0.378143000 | -0.827670000 |
| 1 | 6.351114000 | 1.300570000 | -1.240319000 |
| 1 | 5.095999000 | 0.470102000 | -2.206632000 |
| 6 | 5.104486000 | 1.402314000 | 1.148726000 |
| 1 | 5.516201000 | 0.458424000 | 1.555121000 |
| 1 | 4.401713000 | 1.829460000 | 1.889721000 |
| 1 | 5.948834000 | 2.113067000 | 1.033296000 |

| | | | |
|---|---|---|---|
| 6 | 3.321878000 | -1.152814000 | -0.028734000 |
| 6 | 3.291044000 | -1.921416000 | -1.235198000 |
| 6 | 3.494525000 | -3.313740000 | -1.148773000 |
| 1 | 3.481309000 | -3.915643000 | -2.071426000 |
| 6 | 3.704489000 | -3.947354000 | 0.082089000 |
| 1 | 3.872492000 | -5.035467000 | 0.124741000 |
| 6 | 3.673392000 | -3.192120000 | 1.259485000 |
| 1 | 3.797956000 | -3.698831000 | 2.229651000 |
| 6 | 3.467670000 | -1.795980000 | 1.238038000 |
| 6 | 2.975535000 | -1.319471000 | -2.607742000 |
| 1 | 2.957544000 | -0.216297000 | -2.492699000 |
| 6 | 1.566182000 | -1.749299000 | -3.075573000 |
| 1 | 1.307111000 | -1.253257000 | -4.035065000 |
| 1 | 0.802057000 | -1.474433000 | -2.320194000 |
| 1 | 1.516378000 | -2.847209000 | -3.239808000 |
| 6 | 4.027156000 | -1.676541000 | -3.679628000 |
| 1 | 3.813983000 | -1.135633000 | -4.625700000 |
| 1 | 4.016341000 | -2.761414000 | -3.917038000 |
| 1 | 5.057481000 | -1.418242000 | -3.361365000 |
| 6 | 3.320453000 | -1.071461000 | 2.578313000 |
| 1 | 3.219446000 | 0.011033000 | 2.366306000 |
| 6 | 4.532924000 | -1.274209000 | 3.511580000 |
| 1 | 4.420738000 | -0.661922000 | 4.431038000 |
| 1 | 5.491381000 | -0.991836000 | 3.029702000 |
| 1 | 4.625624000 | -2.332963000 | 3.834123000 |
| 6 | 2.021708000 | -1.516403000 | 3.288583000 |
| 1 | 1.861545000 | -0.923280000 | 4.213804000 |
| 1 | 2.066074000 | -2.588076000 | 3.578661000 |
| 1 | 1.146852000 | -1.369680000 | 2.623515000 |

**3**

$E^{el}_{M06}$ = -2428.9068296

$ZPE_{BP86}$ = 0.799776

E ($E^{el}_{M06}$ + $ZPE_{BP86}$) = -2428.1070536

| | | | |
|---|---|---|---|
| 52 | -2.062399000 | -3.341444000 | 0.222977000 |
| 15 | 0.665382000 | 0.075410000 | 0.146709000 |
| 14 | -1.310831000 | -1.069613000 | 0.061202000 |
| 7 | -2.301371000 | 0.159081000 | 1.121414000 |
| 6 | -2.499657000 | 0.393242000 | 2.574742000 |
| 6 | -2.187167000 | 1.860162000 | 2.947962000 |
| 1 | -2.238854000 | 1.985441000 | 4.049532000 |
| 1 | -2.911133000 | 2.566528000 | 2.496214000 |
| 1 | -1.166112000 | 2.138211000 | 2.615740000 |
| 6 | -1.513451000 | -0.535719000 | 3.314656000 |
| 1 | -1.611899000 | -0.388590000 | 4.409557000 |
| 1 | -0.466125000 | -0.312947000 | 3.025896000 |
| 1 | -1.715740000 | -1.601118000 | 3.081912000 |
| 6 | -3.942244000 | 0.018959000 | 2.986912000 |
| 1 | -4.051787000 | 0.092738000 | 4.089047000 |
| 1 | -4.168722000 | -1.022850000 | 2.681561000 |
| 1 | -4.689723000 | 0.696889000 | 2.529180000 |
| 6 | -2.895694000 | 0.687171000 | 0.031010000 |
| 6 | -3.935828000 | 1.763861000 | 0.018554000 |
| 6 | -5.309305000 | 1.433887000 | 0.030591000 |
| 1 | -5.615390000 | 0.376951000 | 0.062237000 |
| 6 | -6.280751000 | 2.449445000 | 0.008987000 |
| 1 | -7.348940000 | 2.181094000 | 0.020167000 |
| 6 | -5.892103000 | 3.799903000 | -0.025109000 |
| 1 | -6.654982000 | 4.594194000 | -0.042017000 |
| 6 | -4.525791000 | 4.133658000 | -0.037037000 |
| 1 | -4.214882000 | 5.189987000 | -0.064315000 |
| 6 | -3.550619000 | 3.123083000 | -0.014894000 |
| 1 | -2.481187000 | 3.384398000 | -0.029648000 |
| 7 | -2.385515000 | 0.060395000 | -1.047734000 |
| 6 | -2.741498000 | 0.097945000 | -2.485866000 |
| 6 | -4.088944000 | -0.629897000 | -2.707156000 |
| 1 | -4.302635000 | -0.724199000 | -3.792566000 |
| 1 | -4.928334000 | -0.071783000 | -2.245571000 |
| 1 | -4.049121000 | -1.644720000 | -2.260451000 |
| 6 | -2.804310000 | 1.543206000 | -3.026345000 |
| 1 | -2.950091000 | 1.518874000 | -4.126293000 |
| 1 | -1.858599000 | 2.084129000 | -2.817044000 |
| 1 | -3.643416000 | 2.119595000 | -2.591606000 |
| 6 | -1.627006000 | -0.667230000 | -3.229934000 |
| 1 | -1.833481000 | -0.672070000 | -4.319500000 |
| 1 | -1.575994000 | -1.719581000 | -2.882719000 |
| 1 | -0.639218000 | -0.192257000 | -3.060003000 |
| 6 | 2.077738000 | -0.875190000 | -0.311404000 |
| 6 | 2.257789000 | -2.339249000 | -0.763754000 |
| 6 | 3.729404000 | -2.357317000 | -1.269752000 |
| 1 | 4.222233000 | -3.332227000 | -1.078869000 |
| 1 | 3.735909000 | -2.197539000 | -2.368800000 |
| 6 | 4.476009000 | -1.189839000 | -0.591301000 |
| 6 | 1.318879000 | -2.738645000 | -1.918757000 |
| 1 | 1.613534000 | -3.739598000 | -2.301104000 |
| 1 | 1.382489000 | -2.016761000 | -2.759091000 |
| 1 | 0.264796000 | -2.817543000 | -1.584443000 |
| 7 | 3.316133000 | -0.275107000 | -0.259250000 |
| 6 | 2.039145000 | -3.292390000 | 0.437783000 |
| 1 | 2.256245000 | -4.337737000 | 0.131236000 |
| 1 | 0.984817000 | -3.254886000 | 0.780929000 |
| 1 | 2.696284000 | -3.040457000 | 1.294390000 |
| 6 | 5.239139000 | -1.641152000 | 0.672702000 |
| 1 | 5.736590000 | -0.784919000 | 1.168764000 |
| 1 | 6.030034000 | -2.362649000 | 0.380961000 |
| 1 | 4.580650000 | -2.141534000 | 1.408204000 |
| 6 | 5.484088000 | -0.522986000 | -1.542120000 |
| 1 | 5.945801000 | 0.376541000 | -1.085946000 |

| | | | |
|---|---|---|---|
| 1 | 5.016536000 | -0.232564000 | -2.501803000 |
| 1 | 6.297461000 | -1.242871000 | -1.768001000 |
| 6 | 3.520294000 | 1.117754000 | 0.085327000 |
| 6 | 3.761025000 | 1.500951000 | 1.440434000 |
| 6 | 4.074351000 | 2.850674000 | 1.707921000 |
| 1 | 4.272528000 | 3.157493000 | 2.747245000 |
| 6 | 4.120893000 | 3.808550000 | 0.688962000 |
| 1 | 4.375199000 | 4.854950000 | 0.922138000 |
| 6 | 3.815006000 | 3.432784000 | -0.624629000 |
| 1 | 3.813221000 | 4.196417000 | -1.418652000 |
| 6 | 3.501703000 | 2.098576000 | -0.955797000 |
| 6 | 3.601076000 | 0.551644000 | 2.631508000 |
| 1 | 3.415157000 | -0.464081000 | 2.230684000 |
| 6 | 4.848678000 | 0.498346000 | 3.537736000 |
| 1 | 4.713642000 | -0.259748000 | 4.337695000 |
| 1 | 5.029044000 | 1.472505000 | 4.039457000 |
| 1 | 5.769978000 | 0.240395000 | 2.976880000 |
| 6 | 2.356832000 | 0.943367000 | 3.460408000 |
| 1 | 2.177432000 | 0.200947000 | 4.266518000 |
| 1 | 1.456700000 | 0.981739000 | 2.814336000 |
| 1 | 2.486964000 | 1.937525000 | 3.938942000 |
| 6 | 3.085367000 | 1.790989000 | -2.397721000 |
| 1 | 2.991057000 | 0.690133000 | -2.493497000 |
| 6 | 1.692302000 | 2.391323000 | -2.696325000 |
| 1 | 1.358881000 | 2.110606000 | -3.718159000 |
| 1 | 1.714874000 | 3.500985000 | -2.641990000 |
| 1 | 0.942617000 | 2.020150000 | -1.967863000 |
| 6 | 4.114143000 | 2.278019000 | -3.440262000 |
| 1 | 3.820493000 | 1.944439000 | -4.457859000 |
| 1 | 5.134582000 | 1.895393000 | -3.235406000 |
| 1 | 4.174570000 | 3.386593000 | -3.467698000 |

**A**

$E^{el}_{M06}$ = -2160.7454249

$ZPE_{BP86}$ = 0.797234

E ($E^{el}_{M06}$ + $ZPE_{BP86}$) = -2159.9481909

| | | | |
|---|---|---|---|
| 15 | -0.333475000 | 0.059984000 | -0.217004000 |
| 14 | 1.406953000 | 0.056171000 | -1.774303000 |
| 7 | 2.603050000 | 1.134528000 | -0.713834000 |
| 6 | 2.770088000 | 2.580848000 | -0.468100000 |
| 6 | 2.562092000 | 2.895449000 | 1.031940000 |
| 1 | 2.587047000 | 3.992031000 | 1.204155000 |
| 1 | 3.356763000 | 2.437234000 | 1.654402000 |
| 1 | 1.580643000 | 2.506946000 | 1.372552000 |
| 6 | 4.153372000 | 3.088546000 | -0.939388000 |
| 1 | 4.203739000 | 4.194111000 | -0.849332000 |
| 1 | 4.323238000 | 2.821481000 | -2.003019000 |
| 1 | 4.978566000 | 2.664558000 | -0.334508000 |
| 6 | 1.674118000 | 3.291855000 | -1.290754000 |
| 1 | 1.725531000 | 4.388458000 | -1.133287000 |
| 1 | 0.667077000 | 2.939110000 | -0.986548000 |
| 1 | 1.800523000 | 3.089759000 | -2.374910000 |
| 6 | 3.265632000 | 0.054312000 | -0.247306000 |
| 7 | 2.586323000 | -1.027267000 | -0.692575000 |
| 6 | 2.916881000 | -2.467487000 | -0.680285000 |
| 6 | 3.059685000 | -2.991184000 | 0.767382000 |
| 1 | 3.188665000 | -4.093828000 | 0.761419000 |
| 1 | 2.151989000 | -2.751032000 | 1.357274000 |
| 1 | 3.938727000 | -2.553488000 | 1.279766000 |
| 6 | 4.206242000 | -2.755776000 | -1.485972000 |
| 1 | 4.384460000 | -3.849995000 | -1.552103000 |
| 1 | 5.095535000 | -2.298337000 | -1.008456000 |
| 1 | 4.115562000 | -2.356554000 | -2.517376000 |
| 6 | 1.730977000 | -3.188221000 | -1.356713000 |
| 1 | 1.902001000 | -4.283980000 | -1.370591000 |
| 1 | 1.610426000 | -2.843191000 | -2.405327000 |
| 1 | 0.785424000 | -2.981247000 | -0.815488000 |
| 6 | 4.545533000 | 0.056012000 | 0.535521000 |
| 6 | 4.518841000 | -0.033304000 | 1.946194000 |
| 1 | 3.549866000 | -0.106890000 | 2.464150000 |
| 6 | 5.714999000 | -0.028771000 | 2.684069000 |
| 1 | 5.678275000 | -0.096368000 | 3.782991000 |
| 6 | 6.952835000 | 0.060295000 | 2.023726000 |
| 1 | 7.889983000 | 0.061857000 | 2.602710000 |
| 6 | 6.989862000 | 0.148871000 | 0.620689000 |
| 1 | 7.956477000 | 0.219288000 | 0.096818000 |
| 6 | 5.795273000 | 0.149014000 | -0.119083000 |
| 1 | 5.826936000 | 0.222913000 | -1.217272000 |
| 6 | -1.839428000 | -0.026845000 | -1.121781000 |
| 6 | -2.122602000 | -0.017844000 | -2.645621000 |
| 6 | -3.674569000 | 0.093613000 | -2.713436000 |
| 1 | -3.956812000 | 1.157095000 | -2.864319000 |
| 1 | -4.099349000 | -0.480446000 | -3.562437000 |
| 6 | -4.236977000 | -0.381791000 | -1.357239000 |
| 7 | -3.051107000 | -0.122290000 | -0.459846000 |
| 6 | -1.495717000 | 1.207858000 | -3.348915000 |
| 1 | -1.859691000 | 1.263970000 | -4.398161000 |
| 1 | -0.388368000 | 1.138321000 | -3.374017000 |
| 1 | -1.775478000 | 2.150452000 | -2.834644000 |
| 6 | -1.603787000 | -1.309620000 | -3.322705000 |
| 1 | -1.896866000 | -1.322000000 | -4.394616000 |
| 1 | -2.005043000 | -2.225780000 | -2.844452000 |
| 1 | -0.496548000 | -1.353131000 | -3.269260000 |
| 6 | -5.479119000 | 0.421050000 | -0.937131000 |
| 1 | -5.829187000 | 0.135488000 | 0.075657000 |
| 1 | -6.303376000 | 0.214241000 | -1.650665000 |
| 1 | -5.289204000 | 1.510871000 | -0.948703000 |
| 6 | -4.618673000 | -1.878632000 | -1.384140000 |

| | | | |
|---|---|---|---|
| 1 | -4.981175000 | -2.214844000 | -0.393411000 |
| 1 | -3.768769000 | -2.522461000 | -1.682928000 |
| 1 | -5.439835000 | -2.039017000 | -2.113569000 |
| 6 | -3.187905000 | -0.009861000 | 0.976546000 |
| 6 | -3.419391000 | 1.284071000 | 1.541246000 |
| 6 | -3.660968000 | 1.381474000 | 2.926567000 |
| 1 | -3.849118000 | 2.371901000 | 3.370950000 |
| 6 | -3.657606000 | 0.248756000 | 3.749781000 |
| 1 | -3.858834000 | 0.345838000 | 4.828791000 |
| 6 | -3.370041000 | -1.003594000 | 3.195790000 |
| 1 | -3.325171000 | -1.886569000 | 3.853236000 |
| 6 | -3.116115000 | -1.162566000 | 1.816501000 |
| 6 | -3.334087000 | 2.577216000 | 0.726046000 |
| 1 | -3.274079000 | 2.291626000 | -0.344167000 |
| 6 | -2.033054000 | 3.338739000 | 1.066904000 |
| 1 | -1.919793000 | 4.230908000 | 0.414733000 |
| 1 | -1.149616000 | 2.684182000 | 0.924051000 |
| 1 | -2.039595000 | 3.689317000 | 2.121470000 |
| 6 | -4.559856000 | 3.496432000 | 0.910008000 |
| 1 | -4.493956000 | 4.369297000 | 0.226601000 |
| 1 | -4.619427000 | 3.898897000 | 1.943484000 |
| 1 | -5.514738000 | 2.970903000 | 0.704237000 |
| 6 | -2.686953000 | -2.545423000 | 1.320170000 |
| 1 | -2.581015000 | -2.487807000 | 0.218684000 |
| 6 | -3.710502000 | -3.650907000 | 1.654579000 |
| 1 | -3.399340000 | -4.617797000 | 1.205479000 |
| 1 | -4.727671000 | -3.414110000 | 1.280089000 |
| 1 | -3.792704000 | -3.811910000 | 2.750469000 |
| 6 | -1.297625000 | -2.910935000 | 1.892047000 |
| 1 | -0.937820000 | -3.868347000 | 1.458109000 |
| 1 | -1.335632000 | -3.036137000 | 2.995647000 |
| 1 | -0.561074000 | -2.116656000 | 1.651889000 |

**1'**

$E^{el}_{M06}$ = -2558.9648187

$ZPE_{BP86}$ = 0.800264

E ($E^{el}_{M06}$ + $ZPE_{BP86}$) = -2558.1645547

| | | | |
|---|---|---|---|
| 15 | 0.198814000 | -0.494657000 | -0.388766000 |
| 14 | -1.567896000 | -0.494807000 | -1.969848000 |
| 7 | -2.767075000 | -1.271731000 | -0.693997000 |
| 6 | -3.073620000 | -2.640248000 | -0.221008000 |
| 6 | -2.925031000 | -2.735121000 | 1.314850000 |
| 1 | -2.966339000 | -3.797177000 | 1.634860000 |
| 1 | -3.738843000 | -2.194435000 | 1.837014000 |
| 1 | -1.957688000 | -2.294593000 | 1.633424000 |
| 6 | -4.491929000 | -3.070176000 | -0.663239000 |
| 1 | -4.679172000 | -4.125163000 | -0.372735000 |
| 1 | -4.601371000 | -2.990717000 | -1.764968000 |
| 1 | -5.273441000 | -2.446145000 | -0.186185000 |
| 6 | -2.036358000 | -3.570450000 | -0.885316000 |
| 1 | -2.230918000 | -4.624579000 | -0.601561000 |
| 1 | -1.008416000 | -3.306158000 | -0.562383000 |
| 1 | -2.087856000 | -3.497329000 | -1.992396000 |
| 6 | -3.287168000 | -0.067309000 | -0.379723000 |
| 7 | -2.589431000 | 0.848100000 | -1.086971000 |
| 6 | -2.723191000 | 2.315042000 | -1.221602000 |
| 6 | -2.656680000 | 3.007556000 | 0.158490000 |
| 1 | -2.555385000 | 4.104933000 | 0.026091000 |
| 1 | -1.786835000 | 2.627889000 | 0.733184000 |
| 1 | -3.573677000 | 2.824624000 | 0.752062000 |
| 6 | -4.037463000 | 2.679569000 | -1.951564000 |
| 1 | -4.096921000 | 3.776744000 | -2.111283000 |
| 1 | -4.924698000 | 2.377417000 | -1.359830000 |
| 1 | -4.088970000 | 2.182845000 | -2.942845000 |
| 6 | -1.524450000 | 2.775434000 | -2.078258000 |
| 1 | -1.568122000 | 3.871021000 | -2.242820000 |
| 1 | -1.534685000 | 2.279747000 | -3.072458000 |
| 1 | -0.565401000 | 2.536407000 | -1.575321000 |
| 6 | -4.440746000 | 0.199278000 | 0.537899000 |
| 6 | -4.193573000 | 0.463147000 | 1.904320000 |
| 1 | -3.151679000 | 0.465538000 | 2.263475000 |
| 6 | -5.266036000 | 0.715173000 | 2.776074000 |
| 1 | -5.065596000 | 0.919808000 | 3.839739000 |
| 6 | -6.588231000 | 0.706909000 | 2.296271000 |
| 1 | -7.426590000 | 0.905375000 | 2.982870000 |
| 6 | -6.838410000 | 0.444486000 | 0.937810000 |
| 1 | -7.872038000 | 0.436352000 | 0.556909000 |
| 6 | -5.770148000 | 0.190575000 | 0.060166000 |
| 1 | -5.967798000 | -0.016981000 | -1.003162000 |
| 6 | 1.794820000 | -0.411654000 | -1.114133000 |
| 6 | 2.064098000 | -0.873473000 | -2.573695000 |
| 6 | 3.598238000 | -1.120392000 | -2.545652000 |
| 1 | 3.783875000 | -2.188349000 | -2.303868000 |
| 1 | 4.068522000 | -0.918612000 | -3.529636000 |
| 6 | 4.195002000 | -0.246409000 | -1.426850000 |
| 7 | 2.996445000 | -0.103396000 | -0.505776000 |
| 6 | 1.356691000 | -2.191211000 | -2.964393000 |
| 1 | 1.749410000 | -2.546747000 | -3.942041000 |
| 1 | 0.261521000 | -2.054471000 | -3.077633000 |
| 1 | 1.534719000 | -2.982743000 | -2.208352000 |
| 6 | 1.674640000 | 0.226778000 | -3.595138000 |
| 1 | 2.022935000 | -0.056055000 | -4.611848000 |
| 1 | 2.114812000 | 1.211779000 | -3.343240000 |
| 1 | 0.572453000 | 0.348097000 | -3.635133000 |
| 6 | 5.385430000 | -0.939100000 | -0.744479000 |
| 1 | 5.762131000 | -0.353568000 | 0.118000000 |
| 1 | 6.213276000 | -1.033925000 | -1.477175000 |
| 1 | 5.126580000 | -1.955378000 | -0.395125000 |

| | | | |
|---|---|---|---|
| 6 | 4.685873000 | 1.120580000 | -1.952942000 |
| 1 | 5.087594000 | 1.741004000 | -1.129151000 |
| 1 | 3.887879000 | 1.692084000 | -2.463864000 |
| 1 | 5.507984000 | 0.959348000 | -2.680870000 |
| 6 | 3.179894000 | 0.268240000 | 0.885112000 |
| 6 | 3.368287000 | -0.761964000 | 1.858270000 |
| 6 | 3.641440000 | -0.381523000 | 3.187379000 |
| 1 | 3.780608000 | -1.163314000 | 3.950870000 |
| 6 | 3.724541000 | 0.964638000 | 3.562130000 |
| 1 | 3.943953000 | 1.236759000 | 4.607085000 |
| 6 | 3.503367000 | 1.961598000 | 2.605744000 |
| 1 | 3.531618000 | 3.019526000 | 2.911824000 |
| 6 | 3.217935000 | 1.643577000 | 1.261723000 |
| 6 | 3.215636000 | -2.256475000 | 1.553853000 |
| 1 | 3.102905000 | -2.364261000 | 0.454655000 |
| 6 | 1.932200000 | -2.823799000 | 2.203541000 |
| 1 | 1.761595000 | -3.870819000 | 1.873230000 |
| 1 | 1.044905000 | -2.210054000 | 1.947572000 |
| 1 | 2.021120000 | -2.832504000 | 3.311434000 |
| 6 | 4.439051000 | -3.089038000 | 1.996261000 |
| 1 | 4.319664000 | -4.147451000 | 1.681346000 |
| 1 | 4.549037000 | -3.090881000 | 3.101258000 |
| 1 | 5.390909000 | -2.710448000 | 1.571468000 |
| 6 | 2.865654000 | 2.788772000 | 0.309348000 |
| 1 | 2.741520000 | 2.353089000 | -0.701787000 |
| 6 | 3.958829000 | 3.875570000 | 0.238306000 |
| 1 | 3.702891000 | 4.634244000 | -0.531416000 |
| 1 | 4.957643000 | 3.461215000 | -0.010700000 |
| 1 | 4.058282000 | 4.413941000 | 1.204659000 |
| 6 | 1.509973000 | 3.417879000 | 0.699684000 |
| 1 | 1.198625000 | 4.171388000 | -0.055219000 |
| 1 | 1.576518000 | 3.934753000 | 1.681163000 |
| 1 | 0.726055000 | 2.636890000 | 0.787232000 |
| 16 | -0.165107000 | 0.197978000 | 1.479046000 |

**2'**

$E^{el}_{M06} = -4562.2570439$

$ZPE_{BP86} = 0.799357$

$E\ (E^{el}_{M06} + ZPE_{BP86}) = -4561.4576869$

| | | | |
|---|---|---|---|
| 15 | -0.219089000 | -0.493200000 | 0.430633000 |
| 14 | 1.560450000 | -0.564881000 | 1.996694000 |
| 7 | 2.758123000 | -1.301269000 | 0.701902000 |
| 6 | 3.076792000 | -2.659671000 | 0.207044000 |
| 6 | 2.971302000 | -2.724647000 | -1.333666000 |
| 1 | 3.041191000 | -3.779583000 | -1.671989000 |
| 1 | 3.789239000 | -2.159482000 | -1.822566000 |
| 1 | 2.004908000 | -2.296126000 | -1.672091000 |
| 6 | 4.482229000 | -3.100946000 | 0.678567000 |
| 1 | 4.675198000 | -4.150586000 | 0.372848000 |
| 1 | 4.562494000 | -3.043028000 | 1.784176000 |
| 1 | 5.277168000 | -2.469868000 | 0.234171000 |
| 6 | 2.021500000 | -3.602281000 | 0.823902000 |
| 1 | 2.216716000 | -4.649011000 | 0.514487000 |
| 1 | 1.001355000 | -3.323635000 | 0.488824000 |
| 1 | 2.051675000 | -3.560901000 | 1.933507000 |
| 6 | 3.300484000 | -0.091873000 | 0.447307000 |
| 7 | 2.598447000 | 0.803565000 | 1.176405000 |
| 6 | 2.750737000 | 2.260333000 | 1.383578000 |
| 6 | 2.746556000 | 3.016797000 | 0.036745000 |
| 1 | 2.677841000 | 4.109808000 | 0.217631000 |
| 1 | 1.880243000 | 2.695261000 | -0.577159000 |
| 1 | 3.672515000 | 2.829272000 | -0.541224000 |
| 6 | 4.042867000 | 2.568139000 | 2.176540000 |
| 1 | 4.110892000 | 3.655100000 | 2.392720000 |
| 1 | 4.946513000 | 2.282705000 | 1.601517000 |
| 1 | 4.052549000 | 2.022121000 | 3.142894000 |
| 6 | 1.528079000 | 2.702015000 | 2.215845000 |
| 1 | 1.578021000 | 3.789698000 | 2.424998000 |
| 1 | 1.498054000 | 2.168054000 | 3.189621000 |
| 1 | 0.585040000 | 2.493097000 | 1.670996000 |
| 6 | 4.479420000 | 0.195808000 | -0.430727000 |
| 6 | 4.268992000 | 0.497802000 | -1.795313000 |
| 1 | 3.235212000 | 0.513985000 | -2.179229000 |
| 6 | 5.364780000 | 0.769920000 | -2.631288000 |
| 1 | 5.192997000 | 1.004704000 | -3.693760000 |
| 6 | 6.673870000 | 0.743628000 | -2.117327000 |
| 1 | 7.530630000 | 0.957720000 | -2.775971000 |
| 6 | 6.887316000 | 0.443090000 | -0.760476000 |
| 1 | 7.910541000 | 0.420314000 | -0.353005000 |
| 6 | 5.795572000 | 0.169366000 | 0.081575000 |
| 1 | 5.964750000 | -0.068965000 | 1.143381000 |
| 6 | -1.806800000 | -0.438357000 | 1.180726000 |
| 6 | -2.047818000 | -0.935455000 | 2.635642000 |
| 6 | -3.584738000 | -1.165795000 | 2.637161000 |
| 1 | -3.788104000 | -2.225308000 | 2.373503000 |
| 1 | -4.029106000 | -0.983661000 | 3.636849000 |
| 6 | -4.196704000 | -0.257487000 | 1.555556000 |
| 7 | -3.020009000 | -0.112284000 | 0.606404000 |
| 6 | -1.349045000 | -2.272568000 | 2.972748000 |
| 1 | -1.729779000 | -2.652978000 | 3.945726000 |
| 1 | -0.250660000 | -2.152083000 | 3.070661000 |
| 1 | -1.550228000 | -3.038312000 | 2.196215000 |
| 6 | -1.624358000 | 0.131643000 | 3.678131000 |
| 1 | -1.952365000 | -0.177479000 | 4.693835000 |
| 1 | -2.061266000 | 1.126912000 | 3.463908000 |
| 1 | -0.520367000 | 0.241789000 | 3.697394000 |
| 6 | -5.416270000 | -0.913601000 | 0.889052000 |
| 1 | -5.800976000 | -0.304324000 | 0.046766000 |

| | | | |
|---|---|---|---|
| 1 | -6.228424000 | -1.005400000 | 1.639434000 |
| 1 | -5.187527000 | -1.928314000 | 0.515180000 |
| 6 | -4.651487000 | 1.102953000 | 2.129573000 |
| 1 | -5.078518000 | 1.745877000 | 1.336837000 |
| 1 | -3.828863000 | 1.655196000 | 2.622415000 |
| 1 | -5.446192000 | 0.932430000 | 2.885290000 |
| 6 | -3.231545000 | 0.269714000 | -0.778037000 |
| 6 | -3.436092000 | -0.756126000 | -1.753057000 |
| 6 | -3.718116000 | -0.369080000 | -3.078232000 |
| 1 | -3.867878000 | -1.146965000 | -3.843619000 |
| 6 | -3.797891000 | 0.978742000 | -3.447021000 |
| 1 | -4.022963000 | 1.255920000 | -4.489417000 |
| 6 | -3.570385000 | 1.970996000 | -2.487461000 |
| 1 | -3.600965000 | 3.030311000 | -2.788381000 |
| 6 | -3.274145000 | 1.647269000 | -1.147151000 |
| 6 | -3.305974000 | -2.254456000 | -1.454478000 |
| 1 | -3.179768000 | -2.367999000 | -0.357543000 |
| 6 | -2.046591000 | -2.852867000 | -2.122101000 |
| 1 | -1.907703000 | -3.908966000 | -1.805941000 |
| 1 | -1.136255000 | -2.273933000 | -1.865802000 |
| 1 | -2.143147000 | -2.844861000 | -3.229218000 |
| 6 | -4.554572000 | -3.058261000 | -1.880912000 |
| 1 | -4.458599000 | -4.118143000 | -1.562892000 |
| 1 | -4.675541000 | -3.061305000 | -2.984747000 |
| 1 | -5.492161000 | -2.653822000 | -1.448320000 |
| 6 | -2.929895000 | 2.793247000 | -0.191963000 |
| 1 | -2.772792000 | 2.352820000 | 0.812430000 |
| 6 | -4.055065000 | 3.845786000 | -0.091288000 |
| 1 | -3.813038000 | 4.601656000 | 0.685689000 |
| 1 | -5.038409000 | 3.398604000 | 0.162284000 |
| 1 | -4.182092000 | 4.392785000 | -1.049456000 |
| 6 | -1.604362000 | 3.473711000 | -0.600069000 |
| 1 | -1.305751000 | 4.227076000 | 0.160139000 |
| 1 | -1.708184000 | 4.002868000 | -1.571648000 |
| 1 | -0.791762000 | 2.726033000 | -0.715197000 |
| 34 | 0.177820000 | 0.250713000 | -1.577591000 |

**3'**

$E^{el}_{M06}$ = -2428.866354

$ZPE_{BP86}$ = 0.798270

E ($E^{el}_{M06}$ + $ZPE_{BP86}$) = -2428.0680840

| | | | |
|---|---|---|---|
| 15 | -0.241945000 | 0.269565000 | 0.514124000 |
| 14 | 1.581619000 | 1.104275000 | 1.776487000 |
| 7 | 2.699086000 | -0.399536000 | 1.433557000 |
| 6 | 2.956873000 | -1.681020000 | 2.129431000 |
| 6 | 3.070151000 | -2.850595000 | 1.126960000 |
| 1 | 3.097271000 | -3.813654000 | 1.678161000 |
| 1 | 3.994767000 | -2.784903000 | 0.521003000 |
| 1 | 2.199349000 | -2.853411000 | 0.438891000 |
| 6 | 1.738841000 | -1.922211000 | 3.046370000 |
| 1 | 1.867638000 | -2.865785000 | 3.614307000 |
| 1 | 0.806181000 | -1.993691000 | 2.450463000 |
| 1 | 1.623611000 | -1.095872000 | 3.780306000 |
| 6 | 4.235026000 | -1.579854000 | 2.995279000 |
| 1 | 4.379865000 | -2.513320000 | 3.578803000 |
| 1 | 4.161181000 | -0.733696000 | 3.709976000 |
| 1 | 5.136582000 | -1.432458000 | 2.367320000 |
| 6 | 3.363295000 | 0.254599000 | 0.455390000 |
| 6 | 4.591419000 | -0.213820000 | -0.263137000 |
| 6 | 5.882857000 | 0.103058000 | 0.213724000 |
| 1 | 5.996531000 | 0.705284000 | 1.128779000 |
| 6 | 7.021401000 | -0.348668000 | -0.476231000 |
| 1 | 8.024783000 | -0.097649000 | -0.097118000 |
| 6 | 6.879649000 | -1.116966000 | -1.645150000 |
| 1 | 7.772993000 | -1.469600000 | -2.184819000 |
| 6 | 5.595337000 | -1.434650000 | -2.122545000 |
| 1 | 5.479160000 | -2.037208000 | -3.037346000 |
| 6 | 4.453414000 | -0.987621000 | -1.437410000 |
| 1 | 3.437758000 | -1.228874000 | -1.794436000 |
| 7 | 2.733865000 | 1.438656000 | 0.293587000 |
| 6 | 2.992340000 | 2.594848000 | -0.594201000 |
| 6 | 4.324237000 | 3.287309000 | -0.220639000 |
| 1 | 4.462351000 | 4.204825000 | -0.830236000 |
| 1 | 5.192291000 | 2.624627000 | -0.408311000 |
| 1 | 4.328316000 | 3.579855000 | 0.850166000 |
| 6 | 2.999303000 | 2.163797000 | -2.077055000 |
| 1 | 3.063068000 | 3.059673000 | -2.729369000 |
| 1 | 2.072315000 | 1.604217000 | -2.319788000 |
| 1 | 3.866379000 | 1.514946000 | -2.309784000 |
| 6 | 1.828664000 | 3.581149000 | -0.360160000 |
| 1 | 1.953920000 | 4.480766000 | -0.995909000 |
| 1 | 1.796671000 | 3.913060000 | 0.699435000 |
| 1 | 0.857115000 | 3.108392000 | -0.610162000 |
| 6 | -1.820540000 | 0.703431000 | 1.139755000 |
| 6 | -2.007820000 | 1.647978000 | 2.366957000 |
| 6 | -3.546963000 | 1.871102000 | 2.357077000 |
| 1 | -3.949368000 | 2.013818000 | 3.380616000 |
| 1 | -3.774046000 | 2.793711000 | 1.781692000 |
| 6 | -4.185232000 | 0.669172000 | 1.638804000 |
| 6 | -1.317579000 | 3.021987000 | 2.191334000 |
| 1 | -1.668919000 | 3.715833000 | 2.985911000 |
| 1 | -1.560691000 | 3.471747000 | 1.207239000 |
| 1 | -0.214552000 | 2.944138000 | 2.280333000 |
| 7 | -3.060756000 | 0.284620000 | 0.692356000 |
| 6 | -1.521023000 | 1.010165000 | 3.692509000 |
| 1 | -1.803127000 | 1.658092000 | 4.550000000 |
| 1 | -0.415918000 | 0.911592000 | 3.693864000 |
| 1 | -1.951995000 | 0.004455000 | 3.863796000 |

| | | | | | | | |
|---|---|---|---|---|---|---|---|
| 6 | -4.526994000 | -0.459332000 | 2.640177000 | 6 | -4.721930000 | -3.451413000 | 0.691619000 |
| 1 | -5.046552000 | -1.301448000 | 2.147797000 | 1 | -4.630392000 | -4.079672000 | 1.603133000 |
| 1 | -5.207476000 | -0.059109000 | 3.420527000 | 1 | -4.986966000 | -4.127865000 | -0.148506000 |
| 1 | -3.625961000 | -0.854876000 | 3.146774000 | 1 | -5.580281000 | -2.761995000 | 0.834122000 |
| 6 | -5.467117000 | 1.056784000 | 0.888308000 | 6 | -2.265111000 | -3.721235000 | 0.153499000 |
| 1 | -5.866684000 | 0.208552000 | 0.297021000 | 1 | -2.136079000 | -4.378693000 | 1.040000000 |
| 1 | -5.304853000 | 1.910977000 | 0.204306000 | 1 | -1.303593000 | -3.209214000 | -0.057038000 |
| 1 | -6.241168000 | 1.352960000 | 1.625948000 | 1 | -2.492512000 | -4.375406000 | -0.715126000 |
| 6 | -3.333598000 | -0.301166000 | -0.605947000 | 6 | -3.165485000 | 2.075043000 | -1.676281000 |
| 6 | -3.548652000 | -1.705040000 | -0.743265000 | 1 | -3.026268000 | 2.345500000 | -0.609243000 |
| 6 | -3.876913000 | -2.209184000 | -2.018381000 | 6 | -1.867944000 | 2.456650000 | -2.423682000 |
| 1 | -4.036360000 | -3.292358000 | -2.140608000 | 1 | -1.640366000 | 3.534704000 | -2.279991000 |
| 6 | -3.976843000 | -1.371606000 | -3.134462000 | 1 | -1.972549000 | 2.280252000 | -3.515761000 |
| 1 | -4.226122000 | -1.790925000 | -4.122371000 | 1 | -1.003102000 | 1.855279000 | -2.076716000 |
| 6 | -3.746887000 | 0.001330000 | -2.989515000 | 6 | -4.349411000 | 2.904334000 | -2.221576000 |
| 1 | -3.811261000 | 0.655545000 | -3.873454000 | 1 | -4.163086000 | 3.989040000 | -2.072163000 |
| 6 | -3.425982000 | 0.566352000 | -1.739104000 | 1 | -5.309343000 | 2.650409000 | -1.726739000 |
| 6 | -3.399548000 | -2.704465000 | 0.406599000 | 1 | -4.489910000 | 2.745450000 | -3.311482000 |
| 1 | -3.119163000 | -2.127350000 | 1.309616000 | 52 | 0.167352000 | -1.015185000 | -1.501026000 |

## S3. X-Ray Crystallographic Analysis

The data were integrated with SAINT.[12] A multi-scan absorption correction and a 3λ correction[13] was applied using SADABS.[14] The structures were solved by SHELXT[15] and refined on $F^2$ using SHELXL[16] in the graphical user interface ShelXle.[17] All hydrogen atoms were placed using a riding model.

**Table S3.1** Crystal data and structure refinement **1** - **3**.

| Compound | **1** | **2** | **3** |
|---|---|---|---|
| Formula | C35 H54 N3 P S Si | C35 H54 N3 P Se Si | C35 H54 N3 P Si Te |
| Mol. w., g mol$^{-1}$ | 607.93 | 654.83 | 703.47 |
| CCDC no. | 1891853 | 1891854 | 1891855 |
| Wavelength, Å | 0.71073 | 0.71073 | 0.71073 |
| Crystal system | Monoclinic | Monoclinic | Monoclinic |
| Space group | $P2_1/c$ | $P2_1/c$ | $P2_1/c$ |
| *a*, Å | 13.870(3) | 13.903(2) | 35.937(3) |
| *b*, Å | 10.073(2) | 10.077(2) | 9.237(2) |
| *c*, Å | 26.585(3) | 26.651(3) | 23.498(3) |
| *β*,° | 101.65(2) | 102.18(3) | 109.09(2) |
| *V*, Å$^3$ | 3637.7(12) | 3649.8(11) | 7371(2) |
| *Z* | 4 | 4 | 4 |
| *Density* mg/m$^3$ | 1.110 | 1.192 | 1.268 |
| Absorption coefficient, mm$^{-1}$ | 0.192 | 1.131 | 0.909 |
| Crystal size, mm | 0.111 x 0.245 x 0.271 | 0.351 x 0.257 x 0.145 | 0.254 x 0.119 x 0.082 |
| Theta range,° | 2.557 to 27.504 | 1.563 to 28.289 | 0.917 to 25.324 |
| Refl. measured | 54419 | 125128 | 120742 |
| Refl. unique | 8340 | 9105 | 13421 |
| $R_{int}$ | 0.0627 | 0.0424 | 0.0522 |
| Data/ restr./para. | 8340 / 0 / 384 | 9105 / 0 / 384 | 13421 / 0 / 768 |
| $R_1$ [$I$>2σ($I$)][a] | 0.0387 | 0.0252 | 0.0259 |
| $wR_2$ (all refl.)[b] | 0.0973 | 0.0640 | 0.0500 |
| $\Delta\rho_{fin}$, e Å$^{-3}$ | 0.324 and -0.251 | 0.406 and -0.182 | 0.570 and -0.559 |

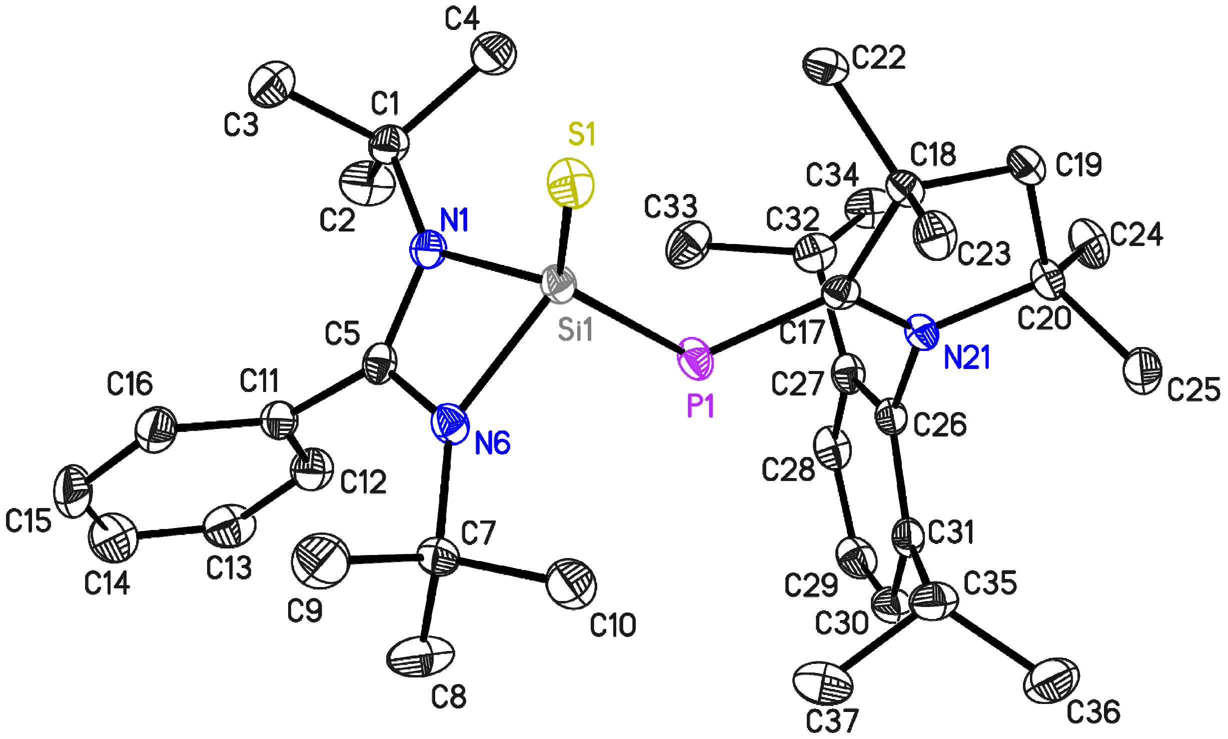


**Figure S3.1** Molecular structure of **1** with thermal ellipsoids at 50% probability level. The hydrogen atoms are omitted for clarity.

**Table S3.2** Bond lengths [Å] and angles [°] for **1**.

| | | | |
|---|---|---|---|
| S(1)-Si(1) | 2.0018(7) | C(1)-C(2) | 1.527(2) |
| P(1)-C(17) | 1.7355(16) | C(5)-N(6) | 1.338(2) |
| P(1)-Si(1) | 2.2433(7) | C(5)-C(11) | 1.490(2) |
| Si(1)-N(6) | 1.8366(14) | N(6)-C(7) | 1.480(2) |
| Si(1)-N(1) | 1.8480(14) | C(7)-C(8) | 1.524(2) |
| Si(1)-C(5) | 2.3013(16) | C(7)-C(9) | 1.524(2) |
| N(1)-C(5) | 1.336(2) | C(7)-C(10) | 1.529(2) |
| N(1)-C(1) | 1.482(2) | C(11)-C(16) | 1.390(2) |
| C(1)-C(3) | 1.525(2) | C(11)-C(12) | 1.395(2) |
| C(1)-C(4) | 1.525(2) | C(12)-C(13) | 1.387(2) |

| | |
|---|---|
| C(13)-C(14) | 1.377(3) |
| C(14)-C(15) | 1.382(3) |
| C(15)-C(16) | 1.390(2) |
| C(17)-N(21) | 1.359(2) |
| C(17)-C(18) | 1.531(2) |
| C(18)-C(22) | 1.528(2) |
| C(18)-C(23) | 1.534(2) |
| C(18)-C(19) | 1.541(2) |
| C(19)-C(20) | 1.528(2) |
| C(20)-N(21) | 1.5032(19) |
| C(20)-C(24) | 1.527(2) |
| C(20)-C(25) | 1.529(2) |
| N(21)-C(26) | 1.4440(19) |
| C(26)-C(31) | 1.404(2) |
| C(26)-C(27) | 1.410(2) |
| C(27)-C(28) | 1.397(2) |
| C(27)-C(32) | 1.526(2) |
| C(28)-C(29) | 1.380(2) |
| C(29)-C(30) | 1.381(2) |
| C(30)-C(31) | 1.397(2) |
| C(31)-C(35) | 1.523(2) |
| C(32)-C(34) | 1.535(2) |
| C(32)-C(33) | 1.536(2) |
| C(35)-C(36) | 1.531(2) |
| C(35)-C(37) | 1.535(3) |
| | |
| C(17)-P(1)-Si(1) | 114.12(6) |
| N(6)-Si(1)-N(1) | 70.96(6) |
| N(6)-Si(1)-S(1) | 117.10(5) |
| N(1)-Si(1)-S(1) | 119.30(5) |
| N(6)-Si(1)-P(1) | 95.38(5) |
| N(1)-Si(1)-P(1) | 99.45(5) |
| S(1)-Si(1)-P(1) | 135.11(3) |
| N(6)-Si(1)-C(5) | 35.52(6) |
| N(1)-Si(1)-C(5) | 35.47(6) |
| S(1)-Si(1)-C(5) | 126.56(5) |
| P(1)-Si(1)-C(5) | 98.02(4) |
| C(5)-N(1)-C(1) | 131.89(13) |
| C(5)-N(1)-Si(1) | 91.13(10) |
| C(1)-N(1)-Si(1) | 136.94(10) |
| N(1)-C(1)-C(3) | 108.56(13) |
| N(1)-C(1)-C(4) | 105.99(12) |
| C(3)-C(1)-C(4) | 109.64(14) |
| N(1)-C(1)-C(2) | 112.57(13) |
| C(3)-C(1)-C(2) | 110.80(14) |
| C(4)-C(1)-C(2) | 109.14(14) |
| N(1)-C(5)-N(6) | 106.27(13) |
| N(1)-C(5)-C(11) | 128.35(14) |
| N(6)-C(5)-C(11) | 125.21(14) |
| N(1)-C(5)-Si(1) | 53.41(8) |
| N(6)-C(5)-Si(1) | 52.92(8) |
| C(11)-C(5)-Si(1) | 173.99(11) |
| C(5)-N(6)-C(7) | 132.00(13) |
| C(5)-N(6)-Si(1) | 91.56(10) |
| C(7)-N(6)-Si(1) | 136.40(10) |
| N(6)-C(7)-C(8) | 112.47(13) |
| N(6)-C(7)-C(9) | 109.11(13) |
| C(8)-C(7)-C(9) | 111.29(16) |
| N(6)-C(7)-C(10) | 105.70(13) |
| C(8)-C(7)-C(10) | 109.00(14) |
| C(9)-C(7)-C(10) | 109.07(15) |
| C(16)-C(11)-C(12) | 120.25(15) |
| C(16)-C(11)-C(5) | 121.39(15) |
| C(12)-C(11)-C(5) | 118.19(14) |
| C(13)-C(12)-C(11) | 119.65(17) |
| C(14)-C(13)-C(12) | 120.20(18) |
| C(13)-C(14)-C(15) | 120.21(17) |
| C(14)-C(15)-C(16) | 120.54(18) |

| | | | |
|---|---|---|---|
| C(15)-C(16)-C(11) | 119.15(17) | C(31)-C(26)-C(27) | 121.35(14) |
| N(21)-C(17)-C(18) | 108.48(13) | C(31)-C(26)-N(21) | 120.68(14) |
| N(21)-C(17)-P(1) | 118.61(11) | C(27)-C(26)-N(21) | 117.97(14) |
| C(18)-C(17)-P(1) | 132.89(12) | C(28)-C(27)-C(26) | 117.81(15) |
| C(22)-C(18)-C(17) | 112.29(13) | C(28)-C(27)-C(32) | 118.39(14) |
| C(22)-C(18)-C(23) | 109.89(13) | C(26)-C(27)-C(32) | 123.71(14) |
| C(17)-C(18)-C(23) | 110.05(13) | C(29)-C(28)-C(27) | 121.41(16) |
| C(22)-C(18)-C(19) | 109.79(13) | C(28)-C(29)-C(30) | 119.72(15) |
| C(17)-C(18)-C(19) | 102.92(12) | C(29)-C(30)-C(31) | 121.42(16) |
| C(23)-C(18)-C(19) | 111.75(13) | C(30)-C(31)-C(26) | 117.86(15) |
| C(20)-C(19)-C(18) | 108.03(12) | C(30)-C(31)-C(35) | 118.23(15) |
| N(21)-C(20)-C(24) | 112.19(13) | C(26)-C(31)-C(35) | 123.65(14) |
| N(21)-C(20)-C(19) | 100.46(12) | C(27)-C(32)-C(34) | 112.38(14) |
| C(24)-C(20)-C(19) | 111.42(14) | C(27)-C(32)-C(33) | 109.88(14) |
| N(21)-C(20)-C(25) | 111.20(13) | C(34)-C(32)-C(33) | 110.20(14) |
| C(24)-C(20)-C(25) | 108.03(14) | C(31)-C(35)-C(36) | 113.35(15) |
| C(19)-C(20)-C(25) | 113.50(13) | C(31)-C(35)-C(37) | 109.33(14) |
| C(17)-N(21)-C(26) | 123.49(13) | C(36)-C(35)-C(37) | 108.97(15) |
| C(17)-N(21)-C(20) | 114.98(12) | | |
| C(26)-N(21)-C(20) | 121.52(12) | | |

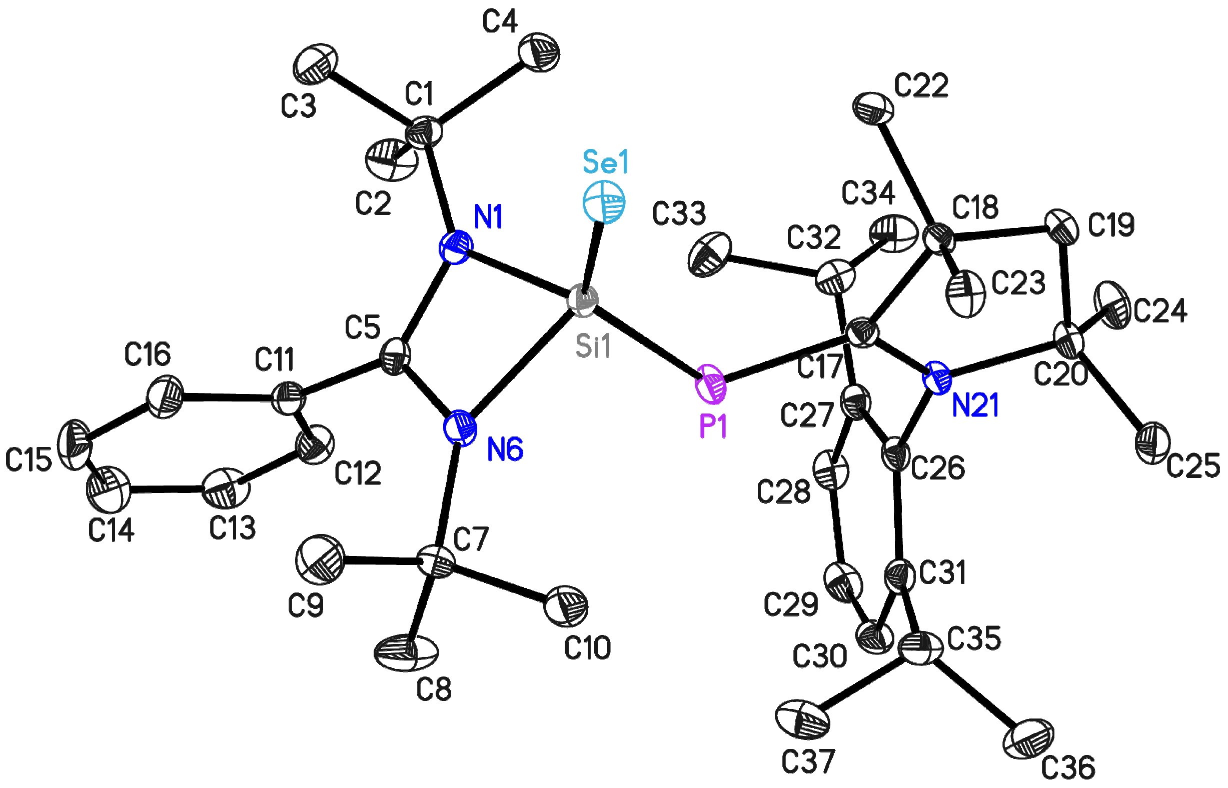


**Figure S3.2** Molecular structure of **2** with thermal ellipsoids at 50% probability level. The hydrogen atoms are omitted for clarity.

**Table S3.2** Bond lengths [Å] and angles [°] for **2.**

| | | | |
|---|---|---|---|
| Se(1)-Si(1) | 2.1404(5) | C(1)-C(3) | 1.5272(18) |
| P(1)-C(17) | 1.7336(12) | C(5)-N(6) | 1.3361(15) |
| P(1)-Si(1) | 2.2384(7) | C(5)-C(11) | 1.4869(17) |
| Si(1)-N(6) | 1.8346(11) | N(6)-C(7) | 1.4769(15) |
| Si(1)-N(1) | 1.8415(12) | C(7)-C(8) | 1.5212(18) |
| Si(1)-C(5) | 2.2969(13) | C(7)-C(9) | 1.5252(18) |
| N(1)-C(5) | 1.3345(15) | C(7)-C(10) | 1.5283(17) |
| N(1)-C(1) | 1.4808(15) | C(11)-C(16) | 1.3872(17) |
| C(1)-C(2) | 1.5231(18) | C(11)-C(12) | 1.3924(18) |
| C(1)-C(4) | 1.5252(17) | C(12)-C(13) | 1.3857(19) |

| | |
|---|---|
| C(13)-C(14) | 1.386(2) |
| C(14)-C(15) | 1.381(2) |
| C(15)-C(16) | 1.3905(19) |
| C(17)-N(21) | 1.3534(15) |
| C(17)-C(18) | 1.5292(16) |
| C(18)-C(23) | 1.5287(16) |
| C(18)-C(22) | 1.5297(16) |
| C(18)-C(19) | 1.5432(16) |
| C(19)-C(20) | 1.5274(17) |
| C(20)-N(21) | 1.5028(15) |
| C(20)-C(24) | 1.5245(17) |
| C(20)-C(25) | 1.5251(17) |
| N(21)-C(26) | 1.4427(15) |
| C(26)-C(31) | 1.4076(17) |
| C(26)-C(27) | 1.4087(17) |
| C(27)-C(28) | 1.3931(17) |
| C(27)-C(32) | 1.5207(17) |
| C(28)-C(29) | 1.3826(19) |
| C(29)-C(30) | 1.3798(19) |
| C(30)-C(31) | 1.3968(18) |
| C(31)-C(35) | 1.5230(18) |
| C(32)-C(34) | 1.5333(19) |
| C(32)-C(33) | 1.5342(18) |
| C(35)-C(36) | 1.5309(19) |
| C(35)-C(37) | 1.5349(19) |
| | |
| C(17)-P(1)-Si(1) | 114.56(4) |
| N(6)-Si(1)-N(1) | 71.05(5) |
| N(6)-Si(1)-Se(1) | 116.49(4) |
| N(1)-Si(1)-Se(1) | 118.40(4) |
| N(6)-Si(1)-P(1) | 95.11(4) |
| N(1)-Si(1)-P(1) | 99.80(4) |
| Se(1)-Si(1)-P(1) | 136.00(2) |
| N(6)-Si(1)-C(5) | 35.56(4) |

| | |
|---|---|
| N(1)-Si(1)-C(5) | 35.51(4) |
| Se(1)-Si(1)-C(5) | 125.36(4) |
| P(1)-Si(1)-C(5) | 98.30(3) |
| C(5)-N(1)-C(1) | 131.79(10) |
| C(5)-N(1)-Si(1) | 91.21(7) |
| C(1)-N(1)-Si(1) | 136.68(8) |
| N(1)-C(1)-C(2) | 112.49(10) |
| N(1)-C(1)-C(4) | 105.98(10) |
| C(2)-C(1)-C(4) | 109.29(11) |
| N(1)-C(1)-C(3) | 108.55(10) |
| C(2)-C(1)-C(3) | 110.65(11) |
| C(4)-C(1)-C(3) | 109.76(11) |
| N(1)-C(5)-N(6) | 106.23(10) |
| N(1)-C(5)-C(11) | 128.05(11) |
| N(6)-C(5)-C(11) | 125.52(11) |
| N(1)-C(5)-Si(1) | 53.28(6) |
| N(6)-C(5)-Si(1) | 52.98(6) |
| C(11)-C(5)-Si(1) | 174.28(9) |
| C(5)-N(6)-C(7) | 132.01(10) |
| C(5)-N(6)-Si(1) | 91.46(8) |
| C(7)-N(6)-Si(1) | 136.49(8) |
| N(6)-C(7)-C(8) | 112.61(10) |
| N(6)-C(7)-C(9) | 109.03(10) |
| C(8)-C(7)-C(9) | 111.14(12) |
| N(6)-C(7)-C(10) | 105.91(9) |
| C(8)-C(7)-C(10) | 108.87(11) |
| C(9)-C(7)-C(10) | 109.09(11) |
| C(16)-C(11)-C(12) | 120.38(12) |
| C(16)-C(11)-C(5) | 121.39(11) |
| C(12)-C(11)-C(5) | 118.10(11) |
| C(13)-C(12)-C(11) | 119.73(12) |
| C(12)-C(13)-C(14) | 120.13(13) |
| C(15)-C(14)-C(13) | 119.87(13) |
| C(14)-C(15)-C(16) | 120.70(13) |

| | | | |
|---|---|---|---|
| C(11)-C(16)-C(15) | 119.19(13) | C(31)-C(26)-C(27) | 121.38(11) |
| N(21)-C(17)-C(18) | 108.49(9) | C(31)-C(26)-N(21) | 120.54(11) |
| N(21)-C(17)-P(1) | 118.49(9) | C(27)-C(26)-N(21) | 118.07(11) |
| C(18)-C(17)-P(1) | 133.00(9) | C(28)-C(27)-C(26) | 117.88(12) |
| C(23)-C(18)-C(17) | 110.12(10) | C(28)-C(27)-C(32) | 118.58(11) |
| C(23)-C(18)-C(22) | 110.05(10) | C(26)-C(27)-C(32) | 123.48(11) |
| C(17)-C(18)-C(22) | 112.17(10) | C(29)-C(28)-C(27) | 121.38(12) |
| C(23)-C(18)-C(19) | 111.70(10) | C(30)-C(29)-C(28) | 119.80(12) |
| C(17)-C(18)-C(19) | 102.87(9) | C(29)-C(30)-C(31) | 121.43(12) |
| C(22)-C(18)-C(19) | 109.76(10) | C(30)-C(31)-C(26) | 117.70(12) |
| C(20)-C(19)-C(18) | 107.97(9) | C(30)-C(31)-C(35) | 118.33(11) |
| N(21)-C(20)-C(24) | 112.30(10) | C(26)-C(31)-C(35) | 123.72(11) |
| N(21)-C(20)-C(25) | 111.32(10) | C(27)-C(32)-C(34) | 112.44(11) |
| C(24)-C(20)-C(25) | 108.29(10) | C(27)-C(32)-C(33) | 109.99(11) |
| N(21)-C(20)-C(19) | 100.22(9) | C(34)-C(32)-C(33) | 109.84(11) |
| C(24)-C(20)-C(19) | 111.31(10) | C(31)-C(35)-C(36) | 113.27(12) |
| C(25)-C(20)-C(19) | 113.34(10) | C(31)-C(35)-C(37) | 109.17(11) |
| C(17)-N(21)-C(26) | 123.53(10) | C(36)-C(35)-C(37) | 109.02(11) |
| C(17)-N(21)-C(20) | 115.24(9) | | |
| C(26)-N(21)-C(20) | 121.23(9) | | |

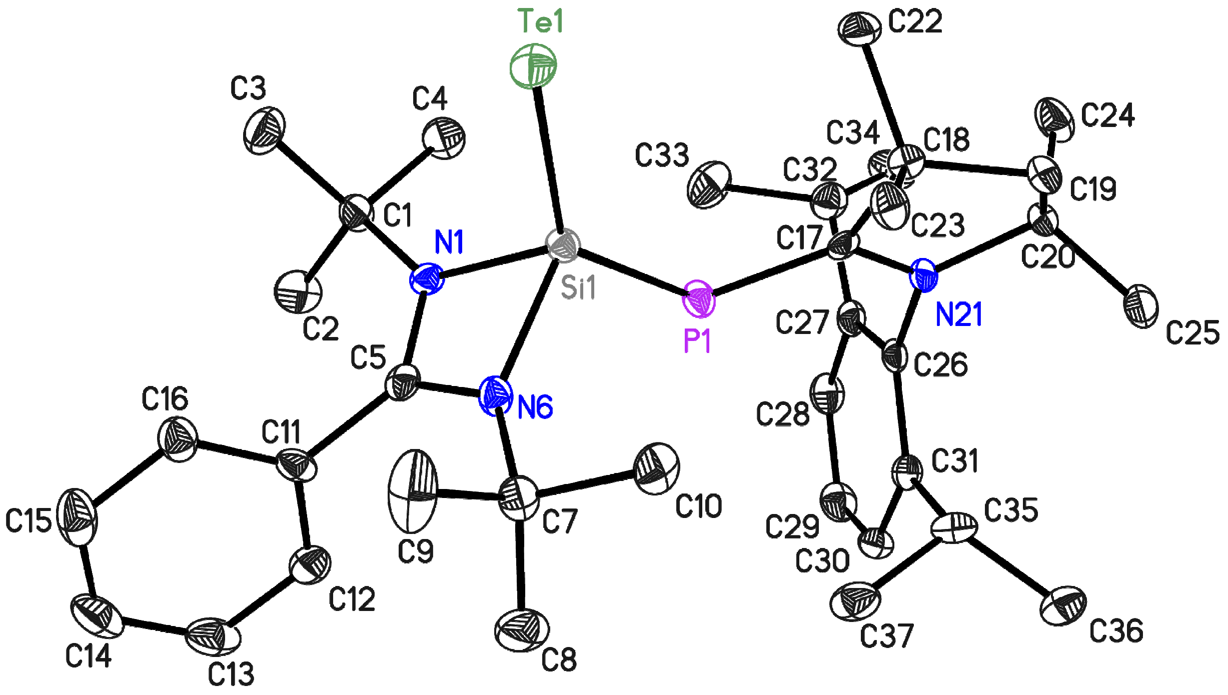


**Figure S3.3** Molecular structure of **3** with thermal ellipsoids at 50% probability level. The hydrogen atoms are omitted for clarity.

The compound crystallizes as a pseudo-merohedral twin with the twin law 1 0 1 0 -1 0 0 0 -1. The fractional contribution of the component refines to 0.5086(4).

**Table S3.2** Bond lengths [Å] and angles [°] for **3.**

| | | | |
|---|---|---|---|
| Te(1)-Si(1) | 2.3766(8) | C(1)-C(3) | 1.527(4) |
| P(1)-C(18) | 1.743(3) | C(1)-C(4) | 1.532(4) |
| P(1)-Si(1) | 2.2395(11) | C(5)-N(12) | 1.331(4) |
| Si(1)-N(1) | 1.829(2) | C(5)-C(6) | 1.484(4) |
| Si(1)-N(12) | 1.845(2) | C(6)-C(7) | 1.391(4) |
| Si(1)-C(5) | 2.300(3) | C(6)-C(11) | 1.394(4) |
| N(1)-C(5) | 1.349(3) | C(7)-C(8) | 1.390(4) |
| N(1)-C(1) | 1.489(3) | C(8)-C(9) | 1.383(5) |
| C(1)-C(2) | 1.511(4) | C(9)-C(10) | 1.386(5) |

| | | | |
|---|---|---|---|
| C(10)-C(11) | 1.377(4) | Si(40)-N(41) | 1.837(2) |
| N(12)-C(13) | 1.490(3) | Si(40)-N(47) | 1.850(2) |
| C(13)-C(14) | 1.518(4) | Si(40)-C(46) | 2.300(3) |
| C(13)-C(15) | 1.518(4) | N(41)-C(46) | 1.336(4) |
| C(13)-C(17) | 1.532(4) | N(41)-C(42) | 1.492(4) |
| C(16)-C(63) | 1.523(4) | C(42)-C(43) | 1.518(4) |
| C(16)-C(58) | 1.538(4) | C(42)-C(45) | 1.520(4) |
| C(16)-C(64) | 1.543(4) | C(42)-C(44) | 1.522(4) |
| C(16)-C(60) | 1.547(4) | C(46)-N(47) | 1.330(4) |
| C(18)-N(22) | 1.362(3) | C(46)-C(52) | 1.497(4) |
| C(18)-C(19) | 1.526(4) | N(47)-C(48) | 1.489(3) |
| C(19)-C(22) | 1.526(4) | C(48)-C(49) | 1.523(4) |
| C(19)-C(23) | 1.537(4) | C(48)-C(51) | 1.524(4) |
| C(19)-C(20) | 1.552(4) | C(48)-C(50) | 1.525(4) |
| C(20)-C(21) | 1.525(4) | C(52)-C(54) | 1.382(4) |
| C(21)-N(22) | 1.503(3) | C(52)-C(53) | 1.387(4) |
| C(21)-C(25) | 1.525(4) | C(53)-C(57) | 1.390(4) |
| C(21)-C(24) | 1.540(4) | C(54)-C(55) | 1.397(4) |
| N(22)-C(26) | 1.438(3) | C(55)-C(56) | 1.369(5) |
| C(26)-C(31) | 1.403(4) | C(56)-C(57) | 1.374(5) |
| C(26)-C(27) | 1.408(4) | C(58)-N(62) | 1.356(3) |
| C(27)-C(28) | 1.390(4) | C(60)-C(61) | 1.526(4) |
| C(27)-C(32) | 1.519(4) | C(61)-N(62) | 1.505(4) |
| C(28)-C(29) | 1.384(4) | C(61)-C(66) | 1.523(4) |
| C(29)-C(30) | 1.373(4) | C(61)-C(65) | 1.530(4) |
| C(30)-C(31) | 1.393(4) | N(62)-C(67) | 1.446(4) |
| C(31)-C(35) | 1.529(4) | C(67)-C(68) | 1.406(4) |
| C(32)-C(33) | 1.536(4) | C(67)-C(72) | 1.412(4) |
| C(32)-C(34) | 1.537(4) | C(68)-C(69) | 1.397(4) |
| C(35)-C(37) | 1.538(4) | C(68)-C(76) | 1.527(4) |
| C(35)-C(36) | 1.538(4) | C(69)-C(70) | 1.377(4) |
| Te(38)-Si(40) | 2.3849(8) | C(70)-C(71) | 1.375(5) |
| P(39)-C(58) | 1.741(3) | C(71)-C(72) | 1.388(4) |
| P(39)-Si(40) | 2.2515(12) | C(72)-C(73) | 1.523(4) |

| | |
|---|---|
| C(73)-C(75) | 1.537(4) |
| C(73)-C(74) | 1.540(4) |
| C(76)-C(78) | 1.537(4) |
| C(76)-C(77) | 1.538(4) |
| | |
| C(18)-P(1)-Si(1) | 114.32(10) |
| N(1)-Si(1)-N(12) | 71.17(10) |
| N(1)-Si(1)-P(1) | 98.62(8) |
| N(12)-Si(1)-P(1) | 104.51(8) |
| N(1)-Si(1)-C(5) | 35.88(10) |
| N(12)-Si(1)-C(5) | 35.35(10) |
| P(1)-Si(1)-C(5) | 105.82(8) |
| N(1)-Si(1)-Te(1) | 112.54(8) |
| N(12)-Si(1)-Te(1) | 114.35(8) |
| P(1)-Si(1)-Te(1) | 135.97(4) |
| C(5)-Si(1)-Te(1) | 117.76(8) |
| C(5)-N(1)-C(1) | 131.6(2) |
| C(5)-N(1)-Si(1) | 91.49(18) |
| C(1)-N(1)-Si(1) | 135.49(18) |
| N(1)-C(1)-C(2) | 112.9(2) |
| N(1)-C(1)-C(3) | 105.6(2) |
| C(2)-C(1)-C(3) | 109.8(3) |
| N(1)-C(1)-C(4) | 108.9(2) |
| C(2)-C(1)-C(4) | 110.9(2) |
| C(3)-C(1)-C(4) | 108.6(2) |
| N(12)-C(5)-N(1) | 105.8(2) |
| N(12)-C(5)-C(6) | 127.3(2) |
| N(1)-C(5)-C(6) | 126.9(3) |
| N(12)-C(5)-Si(1) | 53.32(14) |
| N(1)-C(5)-Si(1) | 52.63(14) |
| C(6)-C(5)-Si(1) | 177.1(2) |
| C(7)-C(6)-C(11) | 119.5(3) |
| C(7)-C(6)-C(5) | 119.7(3) |
| C(11)-C(6)-C(5) | 120.8(3) |

| | |
|---|---|
| C(8)-C(7)-C(6) | 120.0(3) |
| C(9)-C(8)-C(7) | 119.9(3) |
| C(8)-C(9)-C(10) | 120.3(3) |
| C(11)-C(10)-C(9) | 120.1(3) |
| C(10)-C(11)-C(6) | 120.3(3) |
| C(5)-N(12)-C(13) | 130.3(2) |
| C(5)-N(12)-Si(1) | 91.33(17) |
| C(13)-N(12)-Si(1) | 137.91(19) |
| N(12)-C(13)-C(14) | 109.9(2) |
| N(12)-C(13)-C(15) | 111.9(2) |
| C(14)-C(13)-C(15) | 111.4(3) |
| N(12)-C(13)-C(17) | 105.7(2) |
| C(14)-C(13)-C(17) | 109.0(3) |
| C(15)-C(13)-C(17) | 108.7(3) |
| C(63)-C(16)-C(58) | 112.1(2) |
| C(63)-C(16)-C(64) | 110.6(2) |
| C(58)-C(16)-C(64) | 110.1(2) |
| C(63)-C(16)-C(60) | 108.8(2) |
| C(58)-C(16)-C(60) | 102.9(2) |
| C(64)-C(16)-C(60) | 112.1(2) |
| N(22)-C(18)-C(19) | 108.4(2) |
| N(22)-C(18)-P(1) | 118.4(2) |
| C(19)-C(18)-P(1) | 133.2(2) |
| C(18)-C(19)-C(22) | 112.5(2) |
| C(18)-C(19)-C(23) | 110.5(2) |
| C(22)-C(19)-C(23) | 110.1(2) |
| C(18)-C(19)-C(20) | 102.9(2) |
| C(22)-C(19)-C(20) | 108.4(2) |
| C(23)-C(19)-C(20) | 112.4(2) |
| C(21)-C(20)-C(19) | 107.6(2) |
| N(22)-C(21)-C(20) | 100.6(2) |
| N(22)-C(21)-C(25) | 111.9(2) |
| C(20)-C(21)-C(25) | 113.0(3) |
| N(22)-C(21)-C(24) | 111.6(2) |

| | | | |
|---|---|---|---|
| C(20)-C(21)-C(24) | 112.2(2) | C(46)-N(41)-C(42) | 130.9(2) |
| C(25)-C(21)-C(24) | 107.6(2) | C(46)-N(41)-Si(40) | 91.53(18) |
| C(18)-N(22)-C(26) | 121.2(2) | C(42)-N(41)-Si(40) | 135.59(19) |
| C(18)-N(22)-C(21) | 115.0(2) | N(41)-C(42)-C(43) | 105.6(2) |
| C(26)-N(22)-C(21) | 123.7(2) | N(41)-C(42)-C(45) | 112.9(2) |
| C(31)-C(26)-C(27) | 120.8(3) | C(43)-C(42)-C(45) | 110.4(3) |
| C(31)-C(26)-N(22) | 118.5(2) | N(41)-C(42)-C(44) | 108.8(2) |
| C(27)-C(26)-N(22) | 120.6(2) | C(43)-C(42)-C(44) | 109.7(3) |
| C(28)-C(27)-C(26) | 118.3(3) | C(45)-C(42)-C(44) | 109.3(3) |
| C(28)-C(27)-C(32) | 117.5(3) | N(47)-C(46)-N(41) | 106.5(2) |
| C(26)-C(27)-C(32) | 124.2(3) | N(47)-C(46)-C(52) | 126.7(2) |
| C(29)-C(28)-C(27) | 121.3(3) | N(41)-C(46)-C(52) | 126.8(3) |
| C(30)-C(29)-C(28) | 119.8(3) | N(47)-C(46)-Si(40) | 53.53(13) |
| C(29)-C(30)-C(31) | 121.2(3) | N(41)-C(46)-Si(40) | 52.99(14) |
| C(30)-C(31)-C(26) | 118.5(3) | C(52)-C(46)-Si(40) | 177.8(2) |
| C(30)-C(31)-C(35) | 117.9(2) | C(46)-N(47)-C(48) | 130.9(2) |
| C(26)-C(31)-C(35) | 123.6(3) | C(46)-N(47)-Si(40) | 91.14(17) |
| C(27)-C(32)-C(33) | 111.2(3) | C(48)-N(47)-Si(40) | 137.93(18) |
| C(27)-C(32)-C(34) | 110.4(2) | N(47)-C(48)-C(49) | 109.6(2) |
| C(33)-C(32)-C(34) | 109.5(3) | N(47)-C(48)-C(51) | 106.5(2) |
| C(31)-C(35)-C(37) | 111.2(2) | C(49)-C(48)-C(51) | 109.5(2) |
| C(31)-C(35)-C(36) | 110.8(2) | N(47)-C(48)-C(50) | 111.5(2) |
| C(37)-C(35)-C(36) | 110.7(2) | C(49)-C(48)-C(50) | 110.8(3) |
| C(58)-P(39)-Si(40) | 115.04(10) | C(51)-C(48)-C(50) | 108.8(2) |
| N(41)-Si(40)-N(47) | 70.81(10) | C(54)-C(52)-C(53) | 119.9(3) |
| N(41)-Si(40)-P(39) | 96.90(8) | C(54)-C(52)-C(46) | 120.7(3) |
| N(47)-Si(40)-P(39) | 101.26(8) | C(53)-C(52)-C(46) | 119.3(3) |
| N(41)-Si(40)-C(46) | 35.48(10) | C(52)-C(53)-C(57) | 120.0(3) |
| N(47)-Si(40)-C(46) | 35.33(10) | C(52)-C(54)-C(55) | 119.5(3) |
| P(39)-Si(40)-C(46) | 101.03(8) | C(56)-C(55)-C(54) | 120.3(3) |
| N(41)-Si(40)-Te(38) | 114.90(8) | C(55)-C(56)-C(57) | 120.5(3) |
| N(47)-Si(40)-Te(38) | 116.30(8) | C(56)-C(57)-C(53) | 119.8(3) |
| P(39)-Si(40)-Te(38) | 136.56(4) | N(62)-C(58)-C(16) | 108.5(2) |
| C(46)-Si(40)-Te(38) | 122.15(8) | N(62)-C(58)-P(39) | 119.1(2) |

| | |
|---|---|
| C(16)-C(58)-P(39) | 132.3(2) |
| C(61)-C(60)-C(16) | 108.0(2) |
| N(62)-C(61)-C(66) | 112.7(2) |
| N(62)-C(61)-C(60) | 100.9(2) |
| C(66)-C(61)-C(60) | 111.9(3) |
| N(62)-C(61)-C(65) | 111.2(2) |
| C(66)-C(61)-C(65) | 107.9(2) |
| C(60)-C(61)-C(65) | 112.4(2) |
| C(58)-N(62)-C(67) | 123.4(2) |
| C(58)-N(62)-C(61) | 115.0(2) |
| C(67)-N(62)-C(61) | 121.5(2) |
| C(68)-C(67)-C(72) | 121.3(3) |
| C(68)-C(67)-N(62) | 120.5(2) |
| C(72)-C(67)-N(62) | 118.2(3) |
| C(69)-C(68)-C(67) | 117.5(3) |
| C(69)-C(68)-C(76) | 118.5(3) |
| C(67)-C(68)-C(76) | 123.9(3) |
| C(70)-C(69)-C(68) | 121.9(3) |
| C(71)-C(70)-C(69) | 119.6(3) |
| C(70)-C(71)-C(72) | 121.7(3) |
| C(71)-C(72)-C(67) | 118.0(3) |
| C(71)-C(72)-C(73) | 119.2(3) |
| C(67)-C(72)-C(73) | 122.7(3) |
| C(72)-C(73)-C(75) | 112.7(2) |
| C(72)-C(73)-C(74) | 110.4(3) |
| C(75)-C(73)-C(74) | 109.9(2) |
| C(68)-C(76)-C(78) | 110.2(2) |
| C(68)-C(76)-C(77) | 112.2(3) |
| C(78)-C(76)-C(77) | 109.7(3) |

## S4. References

1. S. Kundu, B. Li, J. Kretsch, R. Herbst-Irmer, D. M. Andrada, G. Frenking, D. Stalke, H. W. Roesky, *Angew. Chem. Int. Ed.* **2017**, *56*, 4219–4223; *Angew. Chem.* **2017**, *129*, 4283–4287.

2. a) A. D. Becke, *Phys. Rev. A* **1988**, *38*, 3098–3100; b) J. P. Perdew, *Phys. Rev. B* **1986**, *33*, 8822–8824.

3. F. Weigend, R. Ahlrichs, *Phys. Chem. Chem. Phys.* **2005**, *7*, 3297–3305.

4. Gaussian 09, Revision B.01, M. J. Frisch, G. W. Trucks, H. B. Schlegel, G. E. Scuseria, M. A. Robb, J. R. Cheeseman, G. Scalmani, V. Barone, B. Men- nucci, G. A. Petersson, H.Nakatsuji, M. Caricato, X. Li, H. P. Hratchian, A. F Izmaylov, J. Bloino, G.Zheng, J. L. Sonnenberg, M. Hada, M. Ehara, K. Toyota, R. Fukuda, J. Hasegawa, M. Ishida, T. Nakajima, Y. Honda, O. Ki- tao, H. Nakai, T. Vreven, J. A., Jr., Montgomery, J. E. Peralta, F. Ogliaro, M. Bearpark, J. J. Heyd, E. Brothers, K. N. Kudin, V. N. Staroverov, T. Keith, R. Kobayashi, J. Normand, K.Raghavachari, A. Rendell, J. C. Burant, S. S. Iyengar, J. Tomasi, M. Cossi, N. Rega, J. M. Millam, M. Klene, J. E. Knox, J. B. Cross, V. Bakken, C. Adamo, J. Jaramillo, R. Gomperts,R. E. Strat- mann, O. Yazyev, A. J. Austin, R. Cammi, C. Pomelli, J. W. Ochterski, R. L. Martin, K. Morokuma, V. G. Zakrzewski, G. A. Voth, P. Salvador, J. J. Dan- nenberg, S. Dapprich, A. D. Daniels, O. Farkas, J. B. Foresman, J. V. Ortiz, J. Cioslowski, D. J. Fox, Gaussian, Inc., Wallingford CT, 2010

5. Y. Zhao, D. G. Truhlar, *Theor. Chem. Acc.* **2008**, *120*, 215–241.

6. a) A. E. Reed, L. A. Curtiss, F. Weinhold, *Chem. Rev.* **1988**, *88*, 899–926; b) E. D. Glendening, A. E. Reed, J. E. Carpenter, F. Weinhold, *NBO Version 5.9.*

7. a) T. Lu, F. Chen, *J. Comput. Chem.* **2012**, *33*, 580–592; b) T. Lu, F. Chen, *J. Mol. Graphics Modell.* **2012**, *38*, 314–323.

8. ADF 2016.107, SCM, Theoretical Chemistry, Vrije Universiteit, Amsterdam, http://www.scm.com; G. teVelde, F. M. Bickelhaupt, E. J. Baerends, C. F. Guerra, S. J. A. van Gisbergen, J. G. Snijders, T. Ziegler, *J. Comput. Chem.* **2001**, *22*, 931–967.

9. a) C. Chang, M. Pelissier, P. Durand, *Phys. Scr.* **1986**, *34*, 394–404; b) J. -L. Heully, I. Lindgren, E. Lindroth, S. Lundquist, A.-M. Martensson- Pendrill, *J. Phys. B* **1986**, *19*, 2799–2815; c) E. van Lenthe, E. J. Baerends, J. G. Snijders, *J. Chem. Phys.* **1993**, *99*, 4597–4610; d) E. van Lenthe, J. G. Snijders, E. J. Baerends, *J. Chem. Phys.* **1996**, *105*, 6505– 6516; e) E. van Lenthe, R. van Leeuwen, E. J. Baerends, J. G. Snijders, *Int. J. Quantum Chem.* **1996**, *57*, 281–293; f) E. vanLenthe, E. J. Baerends *J. Comput. Chem.* **2003**, *24*, 1142–1156.

10. a) K. Morokuma, *J. Chem. Phys.* **1971**, *55*, 1236-1244; b) T. Zeigler, A. Rauk, *Inorg. Chem.* **1979**, *18*, 1755-1759; c) T. Zeigler, A. Rauk, *Inorg. Chem.* **1979**, *18*, 1558- 1565; d) M. v. Hopffgarten, G. Frenking, *WIREs Comput. Mol. Sci.* **2012**, *2*, 43-62; e) G. Frenking, F. M. Bickelhaupt in *The Chemical Bond: Fundamental Aspects of Chemical Bonding*, (Ed.: G. Frenking, S. Shaik), Wiley-VCH, Weinheim, Germany, **2014**, Chap. 4, p. 121; f) G. Frenking, R. Tonner, S. Klein, N. Takagi, T. Shimizu, A. Krapp, K. K. Pandey, P. Parameswaran, *Chem. Soc. Rev.* **2014**, *43*, 5106–5139.

11. a)M. Mitoraj, A. Michalak, *Organometallics* **2007**, *26*, 6576-6580; b) M. Mitoraj, A. Michalak, *J, Mol. Model.* **2007**, *13*, 347-355; c) A. Michalak, M. Mitoraj, T. Zeigler, *J. Phys. Chem. A* **2008**, *112*, 1933-1939; d) M. Mitoraj, A. Michalak, *J. Mol. Model.* **2008**, *14*, 681-687; d) M. P. Mitoraj, A. Michalak, T. Ziegler, *J. Chem. Theory Comput.* **2009**, *5*, 962-975; e) T. A. N. Nguyen, G. Frenking, *Chem. Eur. J.* **2012**, *18*, 12733-12748; f) M. Mousavi, G. Frenking, *Organometallics* **2013**, *32*, 1743-1751.
12. Bruker AXS Inc., in *Bruker Apex CCD, SAINT v8.30C* (Ed.: Bruker AXS Inst. Inc.), WI, USA, Madison, 2013.
13. L. Krause, R. Herbst-Irmer, D. Stalke, *J. Appl. Crystallogr.,* **2015**, *48*, 1907-1913.
14. L. Krause, R. Herbst-Irmer, G. M. Sheldrick, D. Stalke, *J. Appl. Crystallogr.* **2015**, *48*, 3-10.
15. G. M. Sheldrick, *Acta Crystallogr.* **2015**, *A71*, 3.
16. G. M. Sheldrick, *Acta Crystallogr.* **2015**, *C71*, 3.
17. C. B. Hübschle, G. M. Sheldrick, B. Dittrich, *J. Appl. Crystallogr.* **2011**, *44*, 1281.
